# Creb3l3 deficiency promotes intestinal lipid accumulation and alters ApoB-containing lipoprotein kinetics

**DOI:** 10.1016/j.jlr.2025.100833

**Published:** 2025-05-29

**Authors:** Darby W. Sweeney, Meng-Chieh Shen, Steven A. Farber

**Affiliations:** Department of Biology, Johns Hopkins University, Baltimore, MD, USA

**Keywords:** CREB3L3, CREBH, enterocyte, chylomicron, zebrafish, lipid

## Abstract

Elevated levels of triglycerides in the bloodstream, a condition known as hypertriglyceridemia, represent a significant risk factor for the development of metabolic disorders and cardiovascular diseases. One key regulator of lipid metabolism is the transcription factor cAMP-responsive element-binding protein 3–like 3 (CREB3L3), which is expressed in the liver, intestine, and adipose tissue. CREB3L3 is localized to the endoplasmic reticulum membrane, and in vertebrates plays a crucial role in plasma lipid homeostasis. However, the precise molecular mechanisms underlying Creb3l3’s influence on cellular lipid metabolism remains undefined. To address this knowledge gap, we generated zebrafish mutants lacking both *creb3l3* orthologs (*creb3l3a* and *creb3l3b*). Gene expression analysis revealed that key *creb3l3* target genes, such as *apoC2* and *apoA4*, were significantly downregulated in the intestines of these double mutants. Using two zebrafish lipoprotein reporter lines, we assessed lipoprotein dynamics in *creb3l3* mutants. Despite producing similar total levels of lipoproteins, *creb3l3* mutants exhibited impaired lipoprotein turnover, suggesting a disruption in circulating lipid clearance. Additionally, histological analysis showed an accumulation of intestinal lipids, characterized by an increased number and size of enterocyte lipid droplets. These findings indicate that *creb3l3* is essential for regulating postprandial lipid flux in enterocytes through altering the balance between lipid storage and secretion. Our study highlights a critical, unappreciated role of Creb3l3 in maintaining intestinal lipid homeostasis.

Dyslipidemia and metabolic syndrome are multifactorial diseases driven by key risk factors such as central adiposity, high blood pressure, insulin resistance, and elevated plasma lipid levels (1). While the intestine is well known for its role in dietary lipid absorption, the molecular processes within enterocytes that influence whole-body lipid physiology remain poorly understood (2, 3, 4). Highly expressed in both the intestine and the liver, the transcription factor, cAMP-responsive element-binding protein 3–like 3 (CREB3L3), is a potential regulator of nutrient absorption and lipid metabolism (5). Humans with heterozygous and homozygous loss-of-function CREB3L3 variants have increased levels of circulating triglycerides (TGs) (6). CREB3L3 is highly conserved from sponges to humans (referred to as CREBH in mouse models), (5) and is tethered to the endoplasmic reticulum (ER) where it remains in an inactive state (Fig. 1). Upon activation, CREB3L3 is transported to the Golgi where the N terminus is then cleaved by site-1 (S1) and site-2 (S2) proteases before being shuttled to the nucleus. This process is similar to that of other important transcription factors known to regulate lipid metabolism such as sterol regulatory element-binding proteins (7, 8). However, the specific molecular players that initiate CREB3L3 cleavage and release from the ER have yet to be elucidated.

**Fig. 1:**
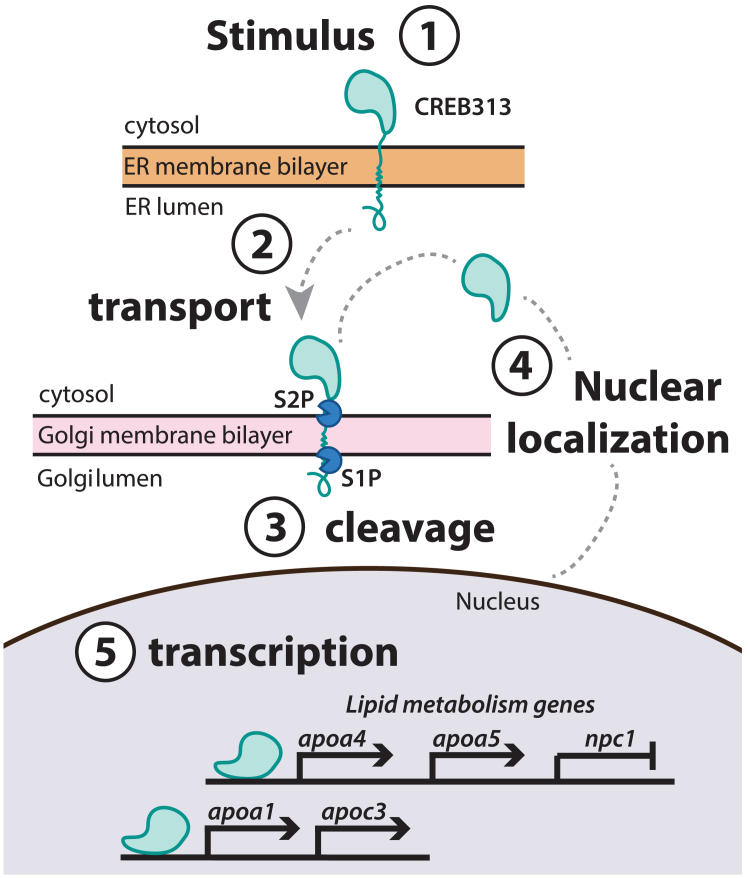
Process of CREB3L3 nuclear localization. 1: In the inactive state, CREB3L3 is anchored to the membrane of the endoplasmic reticulum (ER). 2: An unknown signal induces the release of the tethered protein from the ER membrane, and CREB3L3 is transported to the Golgi. 3: Proteases (S1P, S2P) cleave the transmembrane domain of the protein in the Golgi. 4: Upon cleavage, the DNA-binding domain of the protein can be trafficked to the nucleus. 5: CREB3L3 regulates expression of key lipid metabolism and apoprotein genes by binding to promoter regions and altering transcriptional processes in the nucleus. CREB3L3, cAMP-responsive element-binding protein 3–like 3.

Both the intestine and the liver generate lipoproteins that are responsible for the transport of lipid via the blood stream. Apolipoprotein-B–containing lipoproteins (ApoB-lps) are formed with a single copy of ApoB that provides its structural backbone and is essential for the assembly and secretion of lipoproteins from the intestine (chylomicrons) and liver (VLDLs) (9, 10, 11, 12). The formation of ApoB-lps also requires the activity of microsomal TG transfer protein (MTP). MTP binds the nascent ApoB peptide and transfers lipids from the ER membrane to build the primordial lipoprotein within the ER of enterocytes and hepatocytes (12).

Previous studies of mice null for CREB3L3 (whole body) indicate that they develop and reproduce normally yet have severe hypercholesterolemia and hypertriglyceridemia (13, 14). Further, overexpression of Creb3l3 in hepatocytes improves hypertriglyceridemia and hypercholesterolemia phenotypes in wild-type mouse studies (13, 14). Similarly, intestinal specific overexpression of CREB3L3 in mice fed a lithogenic, or high cholesterol, diet results in a downregulation of intestinal lipid transporters and reduced plasma cholesterol. However, a mechanistic understanding of how CREB3L3 loss impacts lipid metabolism in the intestinal enterocyte remains to be determined.

Using bulk RNA-sequencing (RNA-seq) of the zebrafish gut, we previously identified *creb3l3a* to be significantly upregulated following a high-fat meal (HFM) suggesting that it may regulate postprandial lipid metabolism (15). Here, we further employed the zebrafish model to investigate the impact of Creb3l3 on the balance between intestinal lipid storage and secretion. Using zebrafish at both larval and adult stages, we found that the loss of Creb3l3 results in slower turnover of ApoB-lps in plasma, which is coupled with increased intestinal lipid accumulation, as evidenced by larger and more numerous lipid droplets in enterocytes. These data support a role for Creb3l3 in regulating the balance between intestinal lipid storage and secretion.

## Materials and Methods

### Zebrafish husbandry and maintenance

Zebrafish (*Danio* *rerio*) stock lines were maintained at 27°C in a continuous circulating aquarium facility with a 14:10 h light:dark cycle. Experiments were approved by the Carnegie Institution Department of Embryology Animal Care and Use Committee (Protocol #139). Embryos were collected by natural spawning and raised in petri dishes in embryo media (EM) at 28°C on a 14:10 light: dark cycle until 6 days post fertilization (dpf). At 6 dpf, larvae were moved to the facility on the circulating system and fed a standard diet of GEMMA Micro 75 (Skretting USA) three times per day until 14 dpf. From 15 to 42 dpf, juveniles were fed GEMMA Micro 150 (Skretting USA) three times per day with a supplement of Artemia once daily. Adult zebrafish are fed approximately 3.5% of their individual body weight once daily with Gemma Micro 500 (Skretting USA)—59% protein, 14% lipids, 0.2% fiber, 14% ash, 1.3% phosphorus, 1.5% calcium, 0.7% sodium, 23,000 IU/kg vitamin A, 2800 IU/kg vitamin D3, 1,000 mg/kg vitamin C, 400 mg/kg vitamin E, and supplemented with Artemia.

### In situ hybridization

To generate riboprobes, 866 bp of *creb3l3a* (*ENSDARG00000042332)* and 756 bp of *creb3l3b* (ENSDARG00000013711) mRNA sequences were amplified from cDNA from 6 dpf whole larvae using the primers listed in supplemental Table S3. These amplicons were cloned into the dual promoter plasmid, pCRII-TOPO (Thermo Fisher Scientific, K207020). Sense and antisense probes were synthesized using the DIG RNA labeling kit (Roche 11277073910) using T7 and SP6 polymerases (Roche 10881767001 and 10810274001). Zebrafish larvae at different stages were anesthetized with 0.2% ethyl 3-aminobenzoate methanesulfonate (MS-222) (16) and fixed overnight at 4°C with 4% paraformaldehyde in PBS. Whole-mount in situ hybridization was performed as described by C. Thisse and B. Thisse (17). Larvae were mounted in glycerol and imaged using a Nikon SMZ1500 microscope with HR Plan Apo 1× WD 54 objective, Infinity 3 Lumenera camera, and Infinity Analyze 6.5 software.

### Genome editing with CRISPR to create *creb3l3* mutants

Sequences for *creb3l3a* and *creb3l3b* small guide RNAs (sgRNA) were obtained using CRISPRScan (CRISPR guide sequences are listed in supplemental Table S1). A cloning-free method was used to create template sgRNA as previously described (18). Briefly, gene-specific oligos and an oligo containing a chimeric sgRNA core sequence were synthesized by Eurofins Genomics and were filled in using Phusion polymerase (New England BioLabs) under the following conditions: 98°C for 2 min; 50°C for 10 min; and 72°C for 10 min. Purified PCR product was used as the template DNA for in vitro transcription with the HiScribe T7 High Yield RNA Synthesis kit (New England BioLabs) to generate sgRNA that was purified using phenol: chloroform: isoamyl alcohol (25:24:1) before use. One-cell stage embryos, obtained by natural crosses of wild-type AB zebrafish adults, were injected with 1–2 nl of an injection mix including 500 ng/μl Cas-9 protein (EnGen Spy Cas9 NLS, New England Biolabs #M0646T), sgRNA 300 ng/μl, 300 mM KCl, and 0.4% phenol red. Injected embryos were raised to adulthood (F0) and were crossed to wild-type AB adults for screening. F1 progeny were screened for indels by T7 endonuclease and progeny with promising mutations were outcrossed to wild-type animals to develop stable lines (19). Sequencing was performed to identify the specific mutation present in each line.

### Genotyping *creb3l3* mutants

Genomic DNA was extracted from embryos, larvae, or adult fin clips using a modified version of the PCR-ready DNA extraction protocol (20). Tissue was boiled at 95°C in 50 mM NaOH, cooled, and neutralized with 10% volume of 1 M Tris–HCl pH 8.0. Confirmation of the 2-bp deletion in each gene was performed using PCR primers *creb3l3a_*Fw and *creb3l3a_*Rv for *creb3l3a*^*c802*^ and *creb3l3b_*Fw and *creb3l3*_Rv for *creb3l3b*^*c803*^. Forward primers were designed using dCAPS Finder 2.0 (21) with one mismatch allowing for the wild-type amplicon to code a restriction enzyme site. Primer sequences are listed in supplemental Table S2. Both PCR reactions use an annealing temperature of 60°C and an extension time of 30 s, products are subsequently digested by a unique restriction enzyme for 2 h at 37°C. Following digestion, PCR products are run on a 2% agarose gel in 1X Tris, borate and EDTA buffer. For genotyping *creb3l3a*^*c802*^ animals, the wild-type amplicon is cut by the AvaII restriction enzyme (#R0153S, New England BioLabs) generating a 195-bp product and a 5-bp product that cannot be detected via electrophoresis. *Creb3l3a*^*c802*^ mutant amplicons remain uncut and can be visualized on an agarose gel at 200 bp (supplemental Fig. S1A). The wild-type amplicon for *creb3l3b*^*c803*^ genotyping is digested with MboII (##R0148S, New England BioLabs) to an 80-bp product and a 20-bp product that are both visible on the agarose gel, while the mutant amplicon remains uncut is present at 100-bp (supplemental Fig. S1B).

### Additional zebrafish lines

Both the *ldlra*^*sd52/sd52*^ animals (ZFIN ID: ZDB-ALT-170913-3) and the *apoc2*^*sd38/sd38*^ animals (ZFIN ID: ZDB-ALT-151110-1) were kindly obtained from Y. Miller (UCSD) (22, 23). These lines are maintained as heterozygotes and regularly outcrossed to wild-type animals. Each mutant is genotyped by PCR using established protocols (22, 23). Two different ApoB reporter lines were used in this study, ApoB-NanoLuc (24) and ApoB-Dendra2 (25). Primer sequences for genotyping the ApoB reporter lines are listed in supplemental Table S2. All Dendra2 animals were imaged alongside *apoBb.1*^*+/+*^ animals (not carrying a fluorescent fusion ApoB protein) to control for background fluorescence.

### High-fat feeding

For a high fat meal (HFM), a 5% egg yolk emulsion was prepared following previously described protocols (26, 27). Briefly, 1 ml of frozen chicken egg yolk was thawed to room temperature and mixed with 19 ml EM for a total volume of 20 ml. HFM was made fresh for each experiment. The egg yolk emulsion was pulse sonicated (Sonicator Ultrasonic Processor 6000, Misonix Inc.) with a ¼-inch tapered microtip (4 rounds of 5 s total processing time, 1 s ON, 1 s OFF, output intensity: 3 W). The mixture was strained, vortexed, and kept on ice before use. Larvae at 6 dpf (n = 50) were placed in 35 mm Petri dishes with 5 ml of the egg yolk emulsion and incubated in an orbital shaker (Incu-Shaker Mini H1000-M, Benchmark Scientific) at 29°C, 30 RPM for at 2 h. After feeding, larvae were washed with 1X EM to clear remaining food.

### Oil Red O neutral lipid staining

Zebrafish larvae at different stages (28) (6–14 dpf) were anesthetized with MS-222^16^ before they were fixed with 4% paraformaldehyde in PBS and kept overnight at 4°C with gentle rocking. Fixed fish were washed with 60% 2-propanol for 10 min. Larvae were stained with 0.3% Oil Red O (ORO, Sigma #O0625) dissolved in 60% 2-propanol and rocked overnight at room temperature. Fish were washed with fresh 60% 2-propanol 3 times, rocking at room temperature for 15 min before equilibrating stepwise into 75% glycerol. Larvae were imaged in a final concentration of 75% glycerol using incident light. Images were taken using a Nikon SMZ1500 microscope with HR Plan Apo 1x WD 54 objective, Infinity 3 Lumenera camera, and Infinity Analyze 6.5 software.

### Electron microscopy

Zebrafish larvae fed a HFM were screened for feeding (opaque gut) under a Nikon SMZ-745 stereo microscope. Unfed and fed larvae were anesthetized in 0.2% MS-222^16^ before fixation in 3% glutaraldehyde, 1% formaldehyde, 0.1 M cacodylate solution for 1–3 h. Heads and tails were cut away, and swim bladders were deflated before embedding in 2% low melt agarose. Post fixation was done in 1% osmium tetroxide and 1% potassium ferricyanide in cacodylate solution for 1 h. Following two 10 min washes with NanoPure water, samples were incubated with 0.05 M maleate (pH 6.5) for 10 min. Samples were stained *en bloc* with 0.5% uranyl acetate in maleate for 4°C overnight. Following two 15 min washes with NanoPure water, samples were dehydrated through graded EtOH dilution (35%, 15 min (twice); 50%, 15 min; 75%, 15 min; 95%, 15 min; 100%, 15 min (four times)). Samples were washed with propylene oxide four times for 15 min before incubation with 1:1 propylene oxide/resin (Epon 812 epoxy, Ladd Research Industries, Williston, VT) for 1 h and evaporated overnight. This was followed by two 1 h washes in 100% resin and a final embedding in 100% resin at 55°C overnight followed by 70°C for three days. Sections were made on a Reichert Ultracut-S (Leica Microsystems), mounted on naked 200 thin mesh grids, and stained with lead citrate. Images were obtained with a Phillips Technai-12 electron microscope (FEI, Hillsboro, OR) and 794 Gatan multiscan CCD camera (Gatan, Pleasanton, CA) using Digital Micrograph software.

### RNA extraction and quantitative real-time PCR

After a feeding time course, total RNA was extracted from zebrafish larvae at 6 dpf. A small amount of tail tissue was cut off from the larvae and used for genotyping while the rest of the body was used for RNA extraction. Tissue was homogenized in 200 μl trizol (Invitrogen 15596026) using a Bullet blender storm tissue homogenizer (Next Advance BBY24M) with 0.5 mM zirconium silicate beads (Next Advance ZSB05) at a power of 6 for 1–2 min. RNA was extracted using the Direct-zol RNA microprep kit (Zymo R2061) and eluted in 20 μl of nuclease free water (QIAGEN 129114). cDNA was reverse transcribed using the iScript cDNA synthesis kit (BIO-RAD 1708891) and was used for quantitative RT-PCR (qRT-PCR) with SsoAdvanced Universal SYBR Green Supermix (BIO-RAD). Forward and reverse qRT-PCR primer sequences for each gene were previously validated (15) and are listed in supplemental Table S4. Relative RNA expression level was calculated using the 2-ΔΔCT method using 18S as the reference gene (15, 29).

### Quantification of ApoB-containing ApoB-lps number and size distribution by LipoGlo method

The generation of the LipoGlo reporter (Fus(*apoBb.1*^*NLuc/NLuc*^)) to measure ApoB-lps in the zebrafish and the associated analytical methods and reagents (LipoGlo assays) are described in Thierer, *et al.* (24). *creb3l3a*^*c802*^; *creb3l3b*^*c803*^ mutants were crossed into the LipoGlo background. *Creb3l3* mutant larvae carried one copy of the LipoGlo reporter (*apoBb.1*^*Nluc/+*^) for all LipoGlo experiments.

To quantify ApoB-lps, single larvae were dispensed individually into 96-well plates (USA Scientific, #1402-9589) for homogenization in a total volume of 100 μl of ApoB-lp stabilization buffer (40 mm EGTA, pH 8.0, 20% sucrose, and a cOmplete Mini Protease Inhibitor tablet (Sigma, 11836170001)). Larvae were sonicated in a microplate-horn sonicator (Qsonica Q700 sonicator with a Misonix CL-334 microplate horn assembly). For each sample, an aliquot of 40 μl of homogenate was mixed with 30 μl PBS, 10 μl NanoGlo buffer, and 0.2 μl NanoGlo substrate (Promega, N1110) in a black 96-well plate (Perkin Elmer 6005299), then incubated at room temperature for 5 min. Total luminescence was measured using a BioTek Synergy H1 microplate reader with 500 ms integration or a Tecan Spark microplate reader with 500 ms integration.

For size distribution analyses, ApoB-lps are resolved by running 10 μl of homogenate on a 3% native PAGE gel according to Thierer, *et al.* (24). After electrophoresis, the native gel is incubated with 1 ml of Tris, borate and EDTA buffer and 0.2% Nano-Glo substrate for 3 min and imaged using the Odyssey Fc (Li-Cor Biosciences) gel imaging system. Human LDL-DiI is used as a ladder standard for comparison and its migration distance was used to assign bin cutoffs for zero mobility (ZM), VLDL, intermediate-density lipoprotein (IDL), and LDL fractions (24). Images were analyzed using FIJI software (ImageJ V2.1.0, National Institutes of Health, Bethesda, MD; (140)).

### ApoB-Dendra2 localization and turnover

*creb3l3* mutants were crossed to *apoBb.1*^*Dendra2/Dendra2*^ animals (25). Larvae were mounted in 3% methylcellulose and preconversion images were obtained with an exposure time of 50 ms using the GFP and RFP filters using 20× magnification on an Axio zoom V16 microscope equipped with a Zeiss Plan NeoFluar Z 1x/0.25 FWD 56 mm objective, AxioCam MRm camera, and Zen 2.5 software. The acquisition field was set to 1,388 × 232 pixels. To photoconvert Dendra2, the same live larvae still mounted in methylcellulose were immediately placed under a 385 nm high-power LED light (Thor Labs, SOLIS-385C) for 30 s total at 50% intensity. Post-conversion images were taken directly using both the GFP and RFP filters with an exposure time of 50 ms. After imaging, larvae were immediately moved into individual wells of a 12-well plate containing EM and kept overnight at 28 °C in an incubator with a 14:10 h light:dark cycle. Additional post-conversion images of the same larvae were taken every 24 h. In each experiment, wild-type larvae were imaged each day to control for background fluorescence. Total fluorescence of each image was quantified by raw integrated density in FIJI (ImageJ V2.1.0, National Institutes of Health, Bethesda, MD; (140)). Average values of raw integrated density of background images were subtracted from experimental measurements. Half-life calculations were performed on background subtracted experimental values as previously described (25).

### Adult zebrafish gavage

One microgram of BODIPY FL C12 (Invitrogen, D3822) was resuspended in 5 ml olive oil (Great Value Extra Virgin, Walmart) to a concentration of 75 μg/ml and 1 ml aliquots were stored at −20°C and were protected from light. Adults were anesthetized in 0.4% MS-222^16^ and placed on a wet sponge with fish facility system water to keep gills moist. To gavage, 4 μl of oil was slowly injected into the anterior intestine through the esophageal opening using a 22-gauge Hamilton syringe (Hamilton, Ref: 80665). After gavage, fish were immediately placed in a recovery tank with circulating aquatic facility system water.

### Adult zebrafish blood plasma and tissue collection

Adult zebrafish were euthanized in 0.4% MS-222. Blood is collected from an incision made between the anal fin and the caudal peduncle. Depending on the size and age of adult fish, 5–10 μl of blood is transferred to an Eppendorf tube using EDTA-coated plastic capillary tubes (Kunststoff-Kapillaren 20 μl Innovative Med Tech 100120 or #22-757-123, Thermo Fisher Scientific). Blood is immediately centrifuged at 4°C and at 8,000 RPM for 3 min. The top layer of clear plasma (1–3 μl, typically) is transferred to a fresh Eppendorf tube on ice and diluted 1:100 with ApoB-lp stabilization buffer. Plasma was stored at −20°C with minimal (>1–2) freeze thaw cycles. To quantify BODIPY-C12 fluorescence in plasma, 40 μl of the plasma:buffer dilution was loaded into a 96-well black OptiPlate (PerkinElmer, 6005290). The plate was read immediately after loading by BioTek Synergy H1 microplate reader with an excitation wavelength of 494 and an emission wavelength of 536.

For tissue collection, each animal was dissected on a separate Petri dish cover under a dissecting microscope. The entire intestine, from immediately posterior to the esophagus to the vent, was removed intact. The swim bladder, gall bladder, pancreas, and liver were explicitly removed from the intestine (30, 31). A sterile syringe (Thermo Fisher Scientific, Ref: 14-955-454) was adapted with a 20 μl micropipette tip and the intact intestinal tube was flushed with ∼1 ml 1X PBS to remove any dietary contents trapped in the lumen before the tissue was frozen on dry ice. The intestine was sonicated (Sonicator Ultrasonic Processor 6000, Misonix Inc.) in 1 ml of 1X ApoB-lp stabilization buffer using a ¼ inch tapered microtip (10 s total processing time, 1 s ON, 1 s OFF, output intensity: 3 W). To quantify BODIPY-C12 fluorescence, 50 μl of the intestinal homogenate was loaded into a 96-well black OptiPlate (PerkinElmer, 6005290), and the plate was read immediately using a microplate reader with an excitation wavelength of 494 and an emission wavelength of 536 (BioTek Synergy H1). A Bradford assay (#23246, Thermo Fisher Scientific) was performed to normalize fluorescent signal by total protein (μg) concentration in intestinal homogenate samples calculated based on a standard curve.

### RNA-seq sample preparation and analysis

For larval sequencing, 6 dpf larvae were pooled per *creb3l3* genotype. For adult sequencing, individual fish were fin clipped and genotyped according to protocols described above. Intestinal tissue was extracted as described above (31). The tissue (pooled larvae or single intestine) was homogenized using 0.5 mm zirconium oxide beads (Next Advance, ZrOB05) with a Bullet Blender (Next Advance) in TRIzol reagent (Thermo Fisher Scientific, 15596026). Homogenates were mixed with 100% ethanol 1:1, and RNA was extracted using the Direct-zol RNA Microprep Kit (Zymo Research, R2060). RNA concentration and quality were assessed via the Thermo Fisher Scientific NanoDrop One and the Agilent 2100 Bioanalyzer. RNA-Seq libraries were prepared from approximately 500 ng total RNA using the TruSeq Stranded mRNA Library Prep kit (Illumina, 20020595) and TruSeq RNA CD Index plate (Illumina, 20019792), according to the manufacturer’s protocol. Sequencing was done on the Illumina NextSeq 500, using a 75-bp run with 8×8 indexing, yielding between 20 and 40 million reads per sample. RNA-seq data were processed using the nf–core/rnaseq v3.11.1 pipeline. Briefly, adapter sequences were trimmed from the reads, and ribosomal RNA reads were filtered. The remaining reads were mapped to the GRCz11 genome with Ensembl 110 annotation. Differential expression was examined using the DEseq2 1.42 package for R.

### Measurement of lipid consumption in larvae

Pairwise crosses were performed between *creb3l3a*^*c802/+*^;*creb3l3b*^*c803/+*^ and *creb3l3a*^*c802*^;*creb3l3b*^*c803*^ animals and individual clutches were collected and kept separated. Larvae (6 dpf) of each clutch were fed 0.75 μg/ml BODIPY-C16 (#D3821, Thermo Fisher Scientific) resuspended in 5% egg yolk. The BODIPY-C16 stock is dissolved in ethanol as 0.75 μg/μl. 20 μl of BODIPY stock was added to 20 ml of 5% egg yolk feeding solution. During feeding, fish were incubated at 29°C with 30 RPM shaking for 3 h to promote consumption. After feeding, fish were rinsed three times with EM and sorted by the appearance of a full gut using a lower power stereomicroscope. Fish with empty or much lower than normal food intake were discarded and excluded from sample collection. Fish were then moved to ultra pure water, or double-distilled deionized water (ddH_2_O) and transferred into 96-well nonskirt PCR plate with a total of 25 μl of ddH_2_O. Fish were briefly homogenized with a horn sonicator, and 10 μl of homogenate was separated for downstream genotyping analysis before further sonication. Once genotypes were determined, homogenate of 5 fish of the same genotype were pooled for lipid extraction (around 100 μl total volume). Lipids were extracted using a modified version of the Bligh-Dyer method and spotted on a channeled TLC plate (32, 33). To detect BODIPY-C16, TLC plates were scanned using a Typhoon Biomolecular Imager (Cytiva) using a Cy2 filter. Scanned images were analyzed and quantified with FIJI.

### Statistics

Graphing and some statistics were performed using GraphPad Prism (GraphPad Software). Statistics were also performed using R. Graphical data are presented with mean and SD. Independent technical replicates (N), and sample sizes (n) for each experiment can be found in figure legends or respective text.

## Results

### *creb3l3a* and *creb3l3b* are expressed in zebrafish liver and intestine

**Fig. 2:**
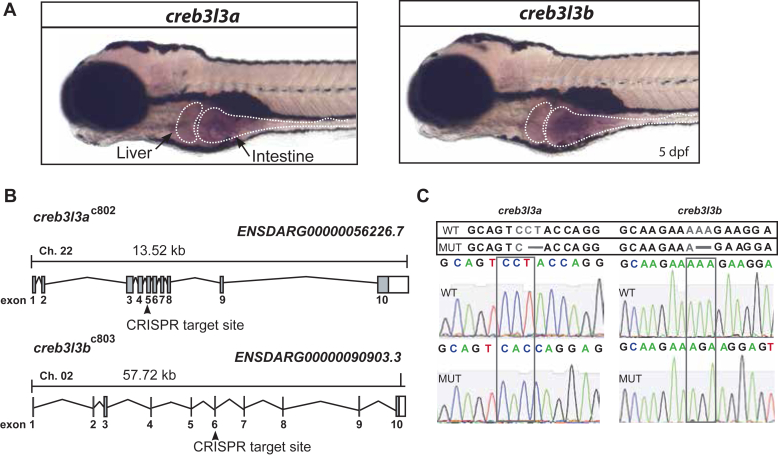
Generation of *creb3l3a* and *creb3l3b* zebrafish mutants. A: Representative images of whole-mount in situ hybridization (ISH) with sense and antisense probes targeting zebrafish *creb3l3a* (ENSDARG00000056226.7) and *creb3l3b* (ENSDARG00000090903.3) at 6 days post fertilization (dpf). Both *creb3l3a* and *creb3l3b* mRNA is expressed in the liver and the intestine at 6 dpf (white outlines). No signal is observed with sense probes. Images are representative of 3 independent clutches: 6–12 animals per clutch. B: Overview of gene loci and strategy used for CRISPR/Cas9 targeting of *creb3l3a* and *creb3l3b*. The fifth exon was targeted by CRISPR guide RNA in *creb3l3a*, and the sixth exon was targeted in *creb3l3b*. C: Sanger sequencing confirms 2-bp deletions in both *creb3l3a* and *creb3l3b* (nucleotides depicted in gray show the affected codon causing a frameshift; dotted box around sequence trace confirms codon change by loss of 2 base pairs). Creb3l3, cAMP-responsive element-binding protein 3–like 3.

We performed whole-mount in situ hybridization to determine the tissue localization of *creb3l3* transcripts in zebrafish larvae. Like in other organisms (34), we found *creb3l3a* and *creb3l3b* expression localized to the liver and the intestine (Fig. 2A). To gain insight into the role of zebrafish *creb3l3* in intestinal lipid metabolism, we generated CRISPR mutants for *creb3l3a* and *creb3l3b* (Fig. 2B). Injected fish were raised to adulthood and outcrossed to wild-type AB fish. Progeny were screened for deleterious insertions or deletions. In the two lines described here, Sanger sequencing confirmed a 2-bp deletion in *creb3l3a* (c.624-625 del, p.Tyr209Profs∗216; allele c802) and a 2bp deletion in *creb3l3b* (c.673-674 del, p.Lys225Glufs∗276; allele c803) (Fig. 2C). The result of these deletions are frame shift mutations predicted to lead to a premature stop codon in the cDNA, likely producing truncated proteins for each gene (supplemental Fig. S2). We created a stable mutant line for *creb3l3a*^*c802*^ and another stable mutant line for *creb3l3b*^*c803*^. Performing a cross of the aforementioned mutant lines, we generated a stable homozygous double mutant: *creb3l3a*^*c802/c802*^;*creb3l3b*^*c803/c803*^. Unless noted otherwise, mutants described below are all double homozygous.

### Lipid accumulation in *creb3l3* mutant intestines

As lecithotrophic animals, larval zebrafish development is dependent upon the metabolism of maternally deposited yolk for up to 5 dpf until the digestive system is sufficiently developed to support food consumption. Before 5 dpf, the normally translucent yolk cell becomes opaque when critical lipid processing genes are disrupted. We have established that this increase in yolk opacity can result from either the accumulation of cytoplasmic lipid droplets or ER lipid retention within the yolk syncytial layer (YSL) (35, 36, 37, 38). For example, MTP mutant animals are unable to efficiently package lipid into ApoB-lps for export from the YSL resulting in abundant YSL cytoplasmic lipid droplets that scatter transmitted light (35). As a result, these animals exhibit a “dark yolk” phenotype. After 5 dpf when the yolk is largely depleted, zebrafish fed a HFM exhibit lipid droplets in their intestinal enterocytes which similarly causes the gut to be opaque when viewed under transmitted light (35).

**Fig. 3:**
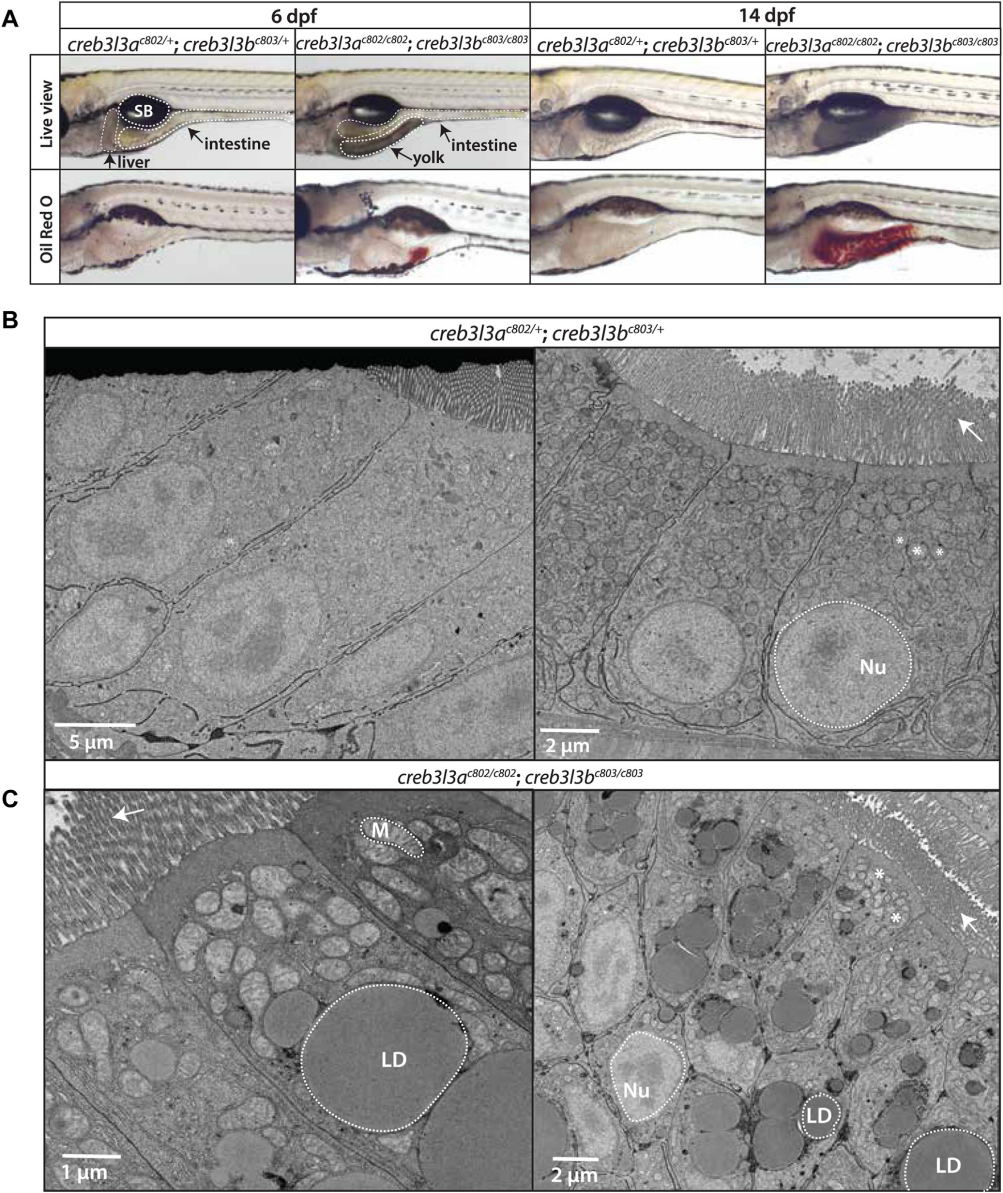
Accumulation of cytoplasmic lipid droplets in the enterocyte of *creb3l3* mutant animals. A: Representative brightfield live images of *creb3l3a*^*c802/+;*^*creb3l3b*^*c803/+*^ and *creb3l3a*^*c802/c802*^*;creb3l3b*^*c803/c803*^ zebrafish larvae at 6 dpf. Unfed 6 dpf *creb3l3* double mutants retain yolk and exhibit a yolk opacity phenotype compared to double heterozygous siblings, which suggests abnormal lipid droplet accumulation in the yolk syncytial layer. *creb3l3a*^*c802/c802*^*;creb3l3b*^*c803/c803*^ animals exhibit abnormal lipid accumulation by Oil Red O (ORO) staining in the posterior region of the yolk at 6 dpf. Images are representative of 3 independent experiments with 5–9 animals per experiment. Fasted 14 dpf *creb3l3* mutants have opaque intestines under brightfield (Live view) and increased ORO staining throughout the intestine in fasted fish at 14 dpf. *creb3l3a*^*c802/+*^*;creb3l3b*^*c803/+*^ animals do not stain with ORO. Images are representative of 3 independent experiments with 5–9 animals per experiment. B: Representative electron microscopy images of *creb3l3a*^*c802/+*^*;creb3l3*^*c803/+*^ and *creb3l3a*^*c802/c802*^*;creb3l3b*^*c803/c803*^ animals fasted at 14 dpf (C). Large lipid droplets (LD) are present in *creb3l3a*^*c802/c802*^*;creb3l3b*^*c803/c803*^ animals. Images are representative of 3–6 animals per genotype. The nucleus and mitochondria are outlined by white dotted lines and lipid droplets are outlined by a solid line. Arrows points to microvilli on the apical side of the enterocyte. Creb3l3, cAMP-responsive element-binding protein 3–like 3; dpf, days post fertilization; Nu, nucleus; M, mitochondria; SB, swim bladder.

In unfed *creb3l3* mutant animals, we observed small portions of remaining yolk at 6 dpf, and the yolk cell had an increased opacity in *creb3l3* double mutants compared to double heterozygote siblings (Fig. 3A). To confirm that the retained yolk is lipid rich, we performed ORO staining which revealed the presence of lipid in unfed *creb3l3* double mutants at 6 dpf (Fig. 3A). The retention of larval yolk could delay or alter normal development. To test if overall growth was altered, we quantified the mass and standard length of *creb3l3* single and double mutants and compared those values to *creb3l3* double heterozygous siblings at 4 months post fertilization (39). Male *creb3l3* mutants weighed slightly less compared to *creb3l3*^*c802/+*^*;creb3l3*^*c803/*^^*+*^ siblings, however no other differences in these developmental markers were observed (supplemental Fig. S3).

To test for the possibility that *creb3l3* mutants accumulated more lipid as a result of consuming more dietary lipid, we fed 6 dpf larvae for 2 h with an HFM of 5% chicken egg yolk spiked with BODIPY-C16 (supplemental Fig. S4A). We quantified the amount of lipid consumption by measuring the total amount of BODIPY fluorescence in whole-body lipid extracts (36, 40). Fish were collected immediately after this meal for lipid extraction. Quantification of BODIPY-C16 revealed no significant differences in the amount of lipid consumed by *creb3l3* mutants (supplemental Fig. S4B). This suggests that the increased retention of intestinal lipid in the *creb3l3* double homozygous mutants does not result from increased dietary lipid consumption.

### Large cytoplasmic lipid droplets in enterocytes of *creb3l3* mutants

To further investigate what may be contributing to the change in intestinal opacity, we turned to juvenile zebrafish that are consuming exogenous meals. *Creb3l3* mutants and control siblings were reared normally and fed a standard diet until 13 dpf. Residual food was cleared from the intestine during a 24-h fasting period. At 14 dpf, we observed dark intestines by low power stereomicroscope in juvenile *creb3l3* mutants (Fig. 3A). The darkened opacity of the intestine indicated a substantial accumulation of lipid that was confirmed by a positive ORO stain (Fig. 3A). Transmission electron microscopy of the enterocytes revealed large cytoplasmic lipid droplets only in *creb3l3* mutants at 14 dpf (Fig. 3C).

### In *creb3l3* mutants, *creb3l3*-responsive genes are not induced following a HFM

**Fig. 4:**
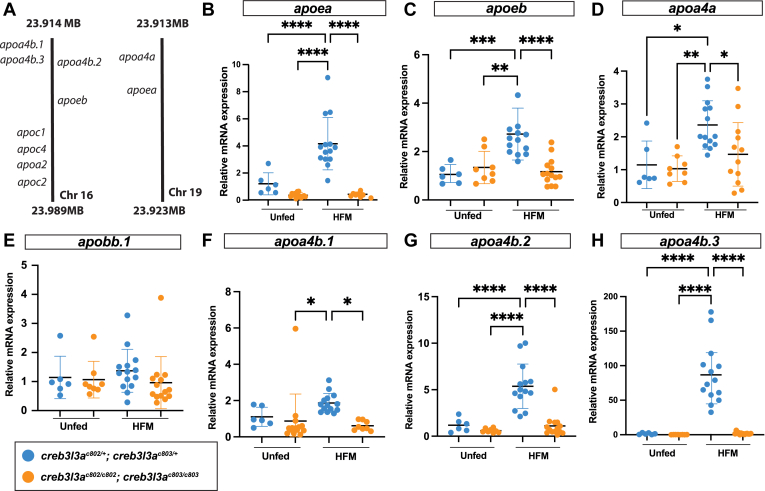
*creb3l3* target genes are not induced in *creb3l3* mutant intestines following a high-fat meal. A: *creb3l3* target genes are clustered together on zebrafish Chr 16 and Chr 19. B: HFM-induced *apoea* expression in *creb3l3a*^*c802/+*^*;creb3l3b*^*c803/+*^ animals, but not in double mutants. Similarly, *apoeb* (C) and *apoa4a* (D) expression increases in *creb3l3a*^*c802/+*^*;creb3l3b*^*c803/+*^ animals after an HFM, but this response is attenuated in *creb3l3a*^*c802/c802*^*;creb3l3b*^*c803/c803*^ animals. E: *apobb.1* expression is not significantly changed between *creb3l3* double heterozygotes and double homozygotes after an HFM. A failure to induce mRNA expression of *apoa4b.1* (F)*, apoa4b.2* (G), and *apoa4b.3* (H) was also noted in *creb3l3* mutants after an HFM. Relative mRNA expression of each gene was normalized to 18S RNA. N = 3 clutches; n = 6–14 individuals per clutch per condition. One way ANOVA with Tukey’s multiple comparisons. ∗∗∗∗*P* < 0.0001, ∗∗∗*P* < 0.001, ∗∗*P* < 0.01, and ∗*P* < 0.05. Creb3l3, cAMP-responsive element-binding protein 3–like 3; HFM, high-fat meal.

Previous work identified *creb3l3a* and other apolipoprotein genes are significantly upregulated in response to a HFM in the zebrafish (15). Interestingly, the physical locations of these apolipoprotein genes on zebrafish chromosomes 16 and 19 are similar to the gene cluster of apolipoproteins observed on human chromosome 11 (Fig. 4A) (41). The apolipoproteins that reside in this gene cluster in humans have been shown to be responsive to Creb3l3 activity (Fig. 4A) (42). We hypothesized that the expression of Creb3l3 target genes (e.g. *apoa4a, apoa4b.1, apoa4b.2, and apoa4b.3)* would also be upregulated following a HFM in zebrafish and that this increase would not occur in the *creb3l3* mutants. To test this, we isolated intestinal tissue from 6 dpf larvae and examined the expression of Creb3l3 target genes in the zebrafish intestine after a HFM using qRT-PCR. Consistent with previous findings, we observed that a HFM induces a transcriptional response of apolipoprotein genes (*apoa4a, apoa4b.1, apoa4b.2, and apoa4b.3)*; however, in *creb3l3* double mutants, the expression of these *creb3l3* target genes is unchanged following the HFM (Fig. 4). Although *apoe* is not a known target of Creb3l3 (43), the expression of both *apoea* and *apoeb* is increased after the consumption of a HFM, but was attenuated in *creb3l3* mutants (Fig. 4B, C).

In rat hepatocytes, the overexpression of CREB3L3 upregulated mRNA and protein levels of apoB (44). Although the promoter of rodent *ApoB* contains a CRE element in which *Creb3l3* can bind (45), the loss of *creb3l3* in the zebrafish does not affect *apoB* gene expression. In *creb3l3* mutants, *apoB* gene expression also remains unchanged in response to a HFM (Fig. 4E). The expression of *apoB* in *creb3l3* mutants likely remains unchanged because *apoB* is constitutively expressed (46).

Expression of *apoa4b.1* is modestly increased in *creb3l3a*^*c802/+*^*;creb3l3b*^*c803/+*^ double heterozygote animals after a HFM, but unchanged in *creb3l3* mutants (Fig. 4F). Similarly, the expression of *apoa4b.2* is significantly increased after a HFM in *creb3l3* double heterozygotes in contrast to *creb3l3* mutants that fail to upregulate *apoa4b.2* expression (Fig. 4G). There is little to no expression of *apoa4b.3* in unfed animals of both genotypes (Fig. 4H), but following a HFM, expression is dramatically increased in the *creb3l3* double heterozygotes and remains unchanged in *creb3l3* mutants (Fig. 4H). While mammals have a single *apoa4* paralog, zebrafish have four *apoa4* genes (47). In zebrafish, *apoa4* gene expression is robustly induced by a HFM, but not in *creb3l3* mutants, paralleling observations in mice where CREB3L3 deficiency leads to lower *ApoA4* mRNA and plasma protein levels, highlighting a conserved role for CREB3L3 in regulating *ApoA4* expression across species.

### Loss of Creb3l3 does not significantly affect ApoB-lp synthesis

**Fig. 5:**
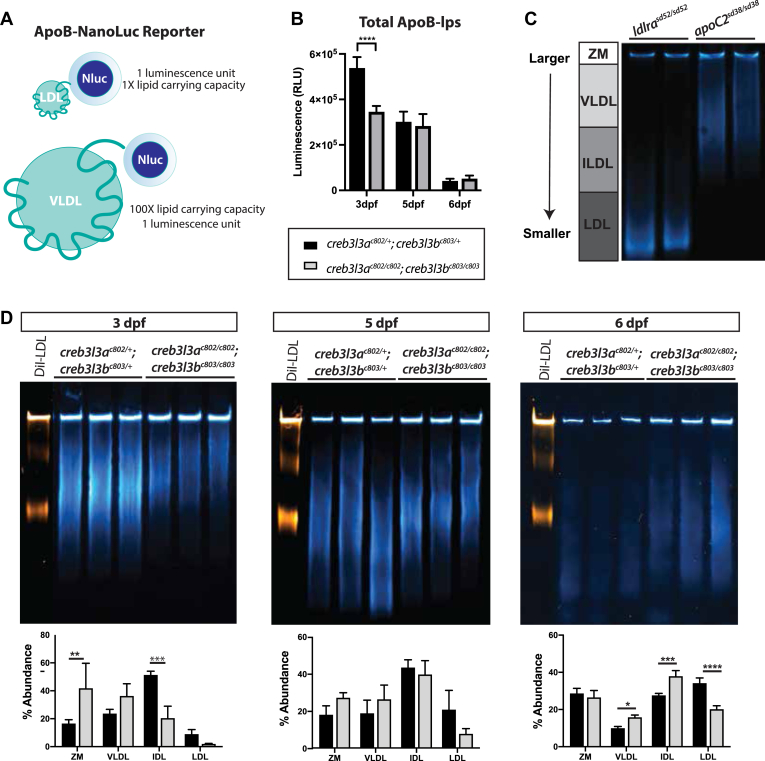
*creb3l3a*^*c802/c802*^*;creb3l3b*^*c803/c803*^ mutants have fewer and larger ApoB-lps at 3 dpf. A: Schematic of LipoGlo reporter on differently sized ApoB-lps. B: Quantification of total ApoB-lps in *creb3l3a*^*c802*^*;creb3l3b*^*c803*^ mutants compared to double heterozygous siblings (*creb3l3a*^*c802/+*^*;creb3l3b*^*c803/+*^) from 3 to 6 dpf. All fish used in these experiments were heterozygous for the ApoBb.1-NanoLuc reporter. Three independent experiments, 8–12 individuals per genotype. One-way ANOVA, ∗∗∗∗*P* < 0.001. C: ApoB-lp particle size distributions of 6 dpf *ldlra*^*sd52*^ mutant and *apoc2*^*sd38*^ mutant homogenate for comparison. D: Native PAGE shows size distribution of ApoB-lp in *creb3l3* double homozygous mutants from 3 to 6 dpf compared to *creb3l3* double heterozygous siblings. Gel images are a composite of chemiluminescent signal (LipoGlo, blue) and fluorescent (DiI-LDL, orange) exposures. DiI-labeled human LDL sample (L3482, Thermo Fisher Scientific) is run in the first lane of each gel as a migration standard. Human Dil-LDL migrates more slowly than NanoLuc-labeled LDL due to the addition of the DiI label. Quantification of luciferase signal on Native PAGE at 3, 5, and 6 dpf by percent abundance of signal within a lane. Gels images are representative of three independent experiments. One-way ANOVA with Tukey’s multiple comparisons. N = 3 clutches; 3–12 individuals per genotype per clutch. ∗∗*P* < 0.01; ∗∗∗*P* < 0.005; and ∗∗∗∗*P* < 0.001. ApoB-lp, apolipoprotein-B–containing lipoprotein; Creb3l3, cAMP-responsive element-binding protein 3–like 3; DiI-LDL, DiI-labeled LDL; dpf, days post fertilization; IDL, intermediate-density lipoprotein; ZM, zero mobility.

ApoB-lps are responsible for lipid transport out of the enterocyte, so we hypothesized that the increase in the number of cytoplasmic lipid droplets in *creb3l3* mutants could be a result of the disruption in the lipid loading of ApoB-lps and/or their secretion from the enterocyte. Although the expression of *apobb.1* is unchanged, we wanted to verify that ApoB-lps are still being secreted. To investigate if the ApoB-lp profile is altered in *creb3l3* mutants, we crossed the *creb3l3a*^*c802/c802*^;*creb3l3b*^*c803/c803*^ mutants to Fus(*apoBb.1*^*NLuc/NLuc*^) transgenic fish (LipoGlo) (24). The LipoGlo reporter line has an in-frame NanoLuciferase sequence inserted at the C terminus of ApoBb.1 (Fig. 5A) (24). A single ApoB molecule is required for ApoB-lp assembly (40), therefore, the LipoGlo reporter can serve as a direct 1:1 readout for the quantity of ApoB-lps in the zebrafish. ApoB-lp levels were measured in larval stages of *creb3l3* mutants throughout development (3–6 dpf). At 3 dpf, there was a significant decrease in the production of ApoB-lps in *creb3l3* double mutants compared to their double heterozygous siblings (Fig. 5B). However, there is no difference in total ApoB-lps between *creb3l3a*^*c802/c802*^;*creb3l3b*^*c803/c803*^ and *creb3l3a*^*c802/+*^;*creb3l3b* ^*c803/+*^ siblings at 5 or 6 dpf (Fig. 5B).

We analyzed the predominate sizes of subclasses of ApoB-Lps using LipoGlo electrophoresis to separate ApoB-lps based on size (24). Genetic mutants that affect ApoB-lp metabolism, such as LDL receptor (*ldlr*) mutants and *apoC2* mutants, are useful standards for assessing particle size. Homogenate samples of these genetic mutants are run on a separate gel side-by-side the *creb3l3* experimental gels to ensure run consistency (Fig. 5C). With the loss of the LDLR, *ldlra*^*sd52/sd52*^ mutants are unable to uptake LDLs, resulting in a distribution of small, dense ApoB-lps (Fig. 5C). In contrast, *apoC2* mutants, *apoc2*^*sd38/sd38*^ lack the cofactor for lipoprotein lipase (LPL) and are unable to release TGs from larger ApoB-lps, resulting in a profile of larger VLDL-sized lipoproteins (Fig. 5C).

Additionally, we use DiI-labeled LDL (DiI-LDL) as a migration standard to ensure consistency between gels. The distance that DiI-LDL migrates down the gel is quantified as one ladder unit (24). It is important to note that human DiI-LDL migrates more slowly than NanoLuc-labeled LDL due to the DiI label. Upon quantification, *creb3l3a*^*c802/c802*^*;creb3l3b*^*c803/c803*^ mutants have larger ApoB-lps in the ZM fraction at 3 dpf. *creb3l3a*^*c802/+*^*;creb3l3b*^*c803/+*^ have statistically more IDL at 3 dpf (Fig. 5D). The ZM fraction is thought to be comprised of larger chylomicrons and ApoB-lp aggregates because this fraction cannot migrate out of the wells. At 3 dpf, *creb3l3* mutants display a lipoprotein profile more similar to that of *apoC2* mutants, although this is only statistically significant in the ZM and IDL fractions. At 5 dpf, there are no statistical differences in ApoB-lp sizes between *creb3l3* genotypes. At 6 dpf, *creb3l3* homozygous double mutants have a higher composition of VLDL and IDL, and less LDL.

### The loss of Creb3l3 affects the lifetime of circulating ApoB-lps

The loss of Creb3l3 results in an accumulation of cytoplasmic lipid droplets in the zebrafish intestine, yet the total amount of ApoB-lps remains unchanged. A number of different mechanisms may underlie this phenotype. ApoB-lps and their remnants undergo lipolysis by LPL, releasing free fatty acids for uptake by peripheral tissues. In addition, hepatic receptors facilitate the uptake and clearance of ApoB-lps from circulation. We hypothesized that slower ApoB-lp turnover may contribute to increased uptake on the basolateral side of intestinal enterocytes, which could contribute to the lipid accumulation phenotype in the intestines of Creb3l3 mutants.

**Fig. 6:**
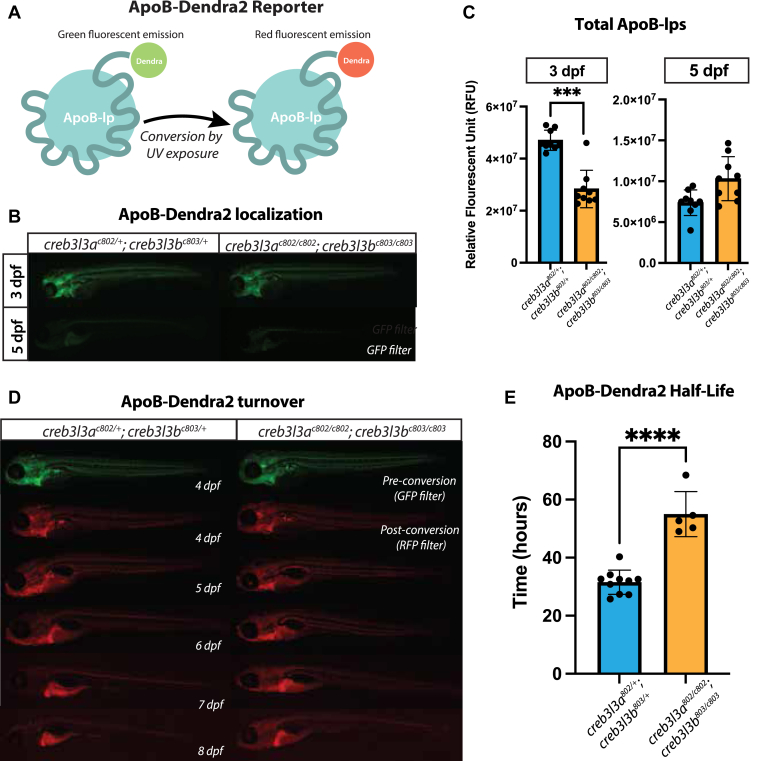
ApoB-Dendra2 reporter localization in *creb3l3* mutants. A: Schematic of ApoB-Dendra2 fusion. Dendra2 is endogenously fused to the C terminus of ApoBb.1 for in vivo characterization of the ApoB-lp profile. The unconverted form of Dendra2 is visualized with a GFP filter. UV exposure irreversibly photoconverts Dendra2 to emit fluorescence visualized by the RFP filter. B: GFP filter showing ApoB-Dendra2 signal in 3 and 5 dpf zebrafish in *creb3l3* double homozygous and double heterozygous siblings. Images are representative of 3 independent clutches; in each clutch, there were 4–10 animals per genotype. All fish used in these experiments were homozygous for the ApoBb.1-Dendra2 reporter and were compared to *apoBb.1*^*+/+*^ for background fluorescence. Unpaired *t*-tests ∗∗∗*P* < 0.0005. C: Quantification of fluorescent signal at 3 and 5 dpf. n = 9–10 animals per genotype. D: Representative images of ApoB-Dendra2 reporter in *creb3l3* double homozygous mutants and *creb3l3* double heterozygous siblings. The same fish is imaged every 24 h from 4 to 8 dpf. Before UV exposure (preconversion) images were taken using the GFP filter and pseudo-colored green. After UV exposure (post conversion) images were taken using an RFP filter and pseudo-colored red. Images are representative of three independent clutches; in each clutch, there were 4–10 animals per genotype. E: Quantification of RFP fluorescent signal from 4 to 8 dpf to measure half-life of ApoB-Dendra2 particles. N = 3 clutches; n = 4–10 individuals per genotype per clutch; unpaired *t*-tests ∗∗∗∗*P* < 0.0001. ApoB-lp, apolipoprotein-B–containing lipoprotein; Creb3l3, cAMP-responsive element-binding protein 3–like 3; dpf, days post fertilization.

Total levels of ApoB-lp can be quantified by both (or either) LipoGlo and LipoTimer transgenic zebrafish (24, 25). LipoTimer employed the same transcription activator-like effector nuclease-mediated genome engineering method that created LipoGlo (24) but replaced the NanoLuciferase sequence with the sequence of the photoconvertible fluorophore, Dendra2. Dendra2 emits light in a green state (ex: 490 nm, em: 553 nm), however, when exposed to UV light, Dendra2 undergoes an irreversible conversion to a red state (ex: 507, em: 573) (Fig. 6A). As ApoB remains with the lipoprotein for its lifetime (48, 49, 50), irreversible photoconversion of the reporter allows for tracking the lifetime of ApoB-lps in the post converted state (25).

To examine ApoB-lp turnover in the absence of Creb3l3, we crossed *creb3l3a*^*c802/c802*^;*creb3l3b*^*c803/c803*^ mutants into the Fus(*apoBb.1*^*Dendra2/Dendra2*^) line and performed whole-body photoconversion experiments. Quantification of the total intracellular and extracellular ApoB-lps green-state fluorescence in live images of *creb3l3* double mutants indicated that at 3 dpf (but not 5 dpf), there was a significant decrease of total ApoB-lps (produced by both the intestine and the liver) in *creb3l3* mutants compared to their double heterozygous siblings (Fig. 6B, C). These data agreed with the ApoB-lps levels determined using the LipoGlo line (Fig. 5B).

To determine whether *creb3l3* mutants exhibit changes in the turnover kinetics of their ApoB-lps, we photoconverted individual fish at 4 dpf and imaged each fish every 24 h from 4 to 8 dpf (Fig. 6D). Quantifying the red-state, postconversion images, we observed that ApoB-lps remain present for a longer amount of time in *creb3l3* mutants than in double heterozygous siblings (Fig. 6E). Our findings demonstrate that the loss of Creb3l3 impairs the clearance of ApoB-lps.

### The loss of Creb3l3 may affect lipid secretion from the enterocyte

To investigate if the loss of Creb3l3 is affecting the secretion rate of lipid from the enterocytes, we gavaged adult zebrafish with a fluorescent lipid analog (BODIPY-FL C12). In mice, quantification of intestinal lipid secretion is typically performed in the presence of a pharmacologic lipase inhibitor, Poloxamer, which prevents ApoB-lps from being lipolyzed by LPL. Thus, quantitation of tracer lipids in the plasma is not influenced by lipoprotein turnover and clearance. Due to the challenges associated with drug delivery in the larval zebrafish, we utilized a genetic mutant deficient in lipolysis, *apoC2*^*sd38*^, to aid in our evaluation of the rate of intestinal lipid secretion. *ApoC2*^*sd38*^ animals lack the cofactor (ApoC2) needed for LPL to dock on the particle, thus in *apoC2*^*sd38*^ mutants, lipoprotein hydrolysis is blocked. Following a meal, dietary lipids administered via gavage are packaged into ApoB-lps and secreted from enterocytes. However, in the absence of ApoC2, ApoB-lps cannot be lipolyzed by LPL and consequently remain in circulation.

**Fig. 7:**
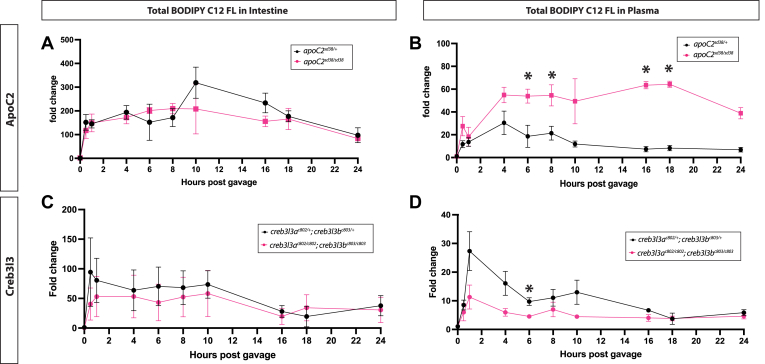
The loss of Creb3l3 may affect lipid secretion from the enterocyte. A: Fluorescent signal of BODIPY-C12 within intestinal homogenate over 24 h post gavage shows no significant difference between *apoC2*^*sd38*^ animals and *apoC2*^*sd38/+*^ control animals. B: 24-h postprandial trend of BODIPY-FL C12 fluorescent signal in blood plasma. Fluorescent signal in *apoC2*^*sd38*^ animals remains elevated from 4 h to 24 h post gavage compared to *apoC2*^*sd38/+*^ animals. C: Fluorescent signal within intestinal homogenate over 24 h post gavage. *Creb3l3* mutants show slightly less total fluorescence over time, yet there is no significant difference between *creb3l3* double homozygous mutants and *creb3l3* double heterozygote control animals. D: Blood plasma BODIPY-FL C12 fluorescent signal is reduced in *creb3l3* double homozygous mutants and significantly different at 6 h post gavage. For each experiment, the mean fluorescent signal at each time point was divided by the mean fluorescent value of the control (heterozygotes) at t = 0 to calculate the fold change between the individual genotypes. The average fold change for each experiment was plotted. n = 3 individual fish of each genotype at each time point for each experiment; three independent experiments for each time course. ∗ = *P* < 0.05, Two-way ANOVA with multiple comparisons. Creb3l3, cAMP-responsive element-binding protein 3–like 3.

We first analyzed BODIPY-FL C12 fluorescence in the intestine and in the plasma of *apoC2*^*sd38*^ mutants and siblings following gavage (Fig. 7A, B). After a gavage, BODIPY-FL C12 fluorescent signal in the plasma of *apoC2*^*sd38/+*^ control siblings is high following the gavage but begins to be cleared 4 h after gavage (Fig. 7B). In contrast, BODIPY-FL C12 fluorescent signal remained significantly elevated from 6 to 18 h in the plasma of *apoC2*^*sd38*^ mutants (Fig. 7B). LPL is active in circulation, outside of the intestinal cells, so it was not surprising that we saw no significant differences of BODIPY-FL C12 fluorescent signal in intestinal tissue of *apoc2* mutants (Fig. 7A).

We next performed the same gavage and analysis in *creb3l3* mutant adults and double heterozygous siblings. We found there was no significant difference in BODIPY-FL C12 fluorescence in intestinal tissue in mutants after gavage compared to control animals (Fig. 7C). However, in circulation, we observe significantly less BODIPY-FL C12 in *creb3l3* mutants at 6 h post gavage (Fig. 7D). The reduced levels of circulating lipids suggest that *creb3l3* double mutants secrete lipids less efficiently than *creb3l3* double heterozygotes.

### RNA-seq analysis confirms gene expression shifts in both larvae and adults

We conducted RNA-seq at different stages in fasted and postprandial states to investigate potential differences in gene regulatory networks resulting from the loss of Creb3l3. RNA was collected from both larval animals (6 dpf; fed and unfed) and from isolated adult intestinal tissue (<1 years old; fed and unfed) to interrogate changes in intestinal gene expression.

**Fig. 8:**
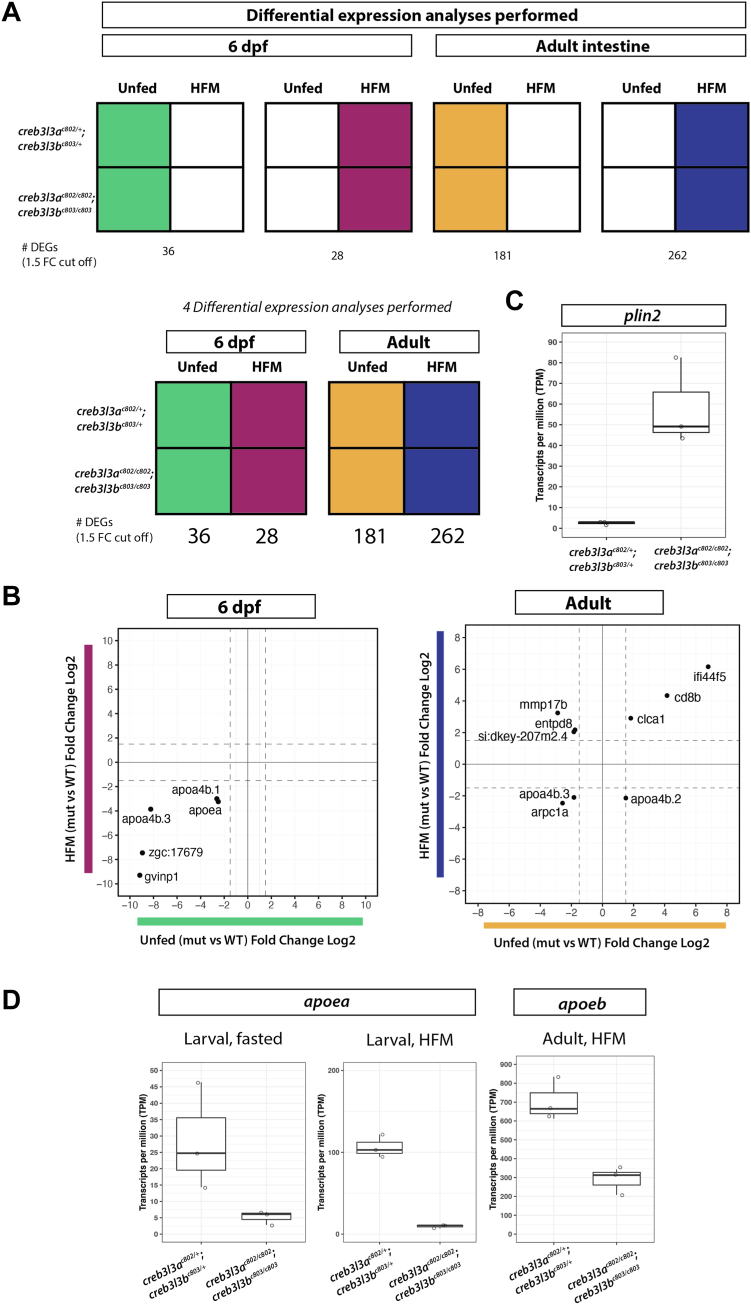
RNA sequencing analysis of *creb3l3* mutants at larval and adult stages. A: Visual description of the 4 different analyses performed. Using a cut off of 1.5 for log2 fold change (FC), 6 dpf unfed *creb3l3* double mutants have 36 differentially expressed genes (DEGs) compared to double heterozygous siblings (green analysis). After a high-fat meal (HFM), 6 dpf *creb3l3* double mutants have 28 DEGs (pink). The number of DEGs significantly increases in adult animals. In the unfed state, *creb3l3* double mutant adults have 181 DEGs (orange), and after an HFM, *creb3l3* adults had 262 DEGs compared to double heterozygous siblings (blue analysis). B: The lists of DEGs between each feeding condition were compared for each age group and cross plotted (green versus pink and orange versus blue). In 6 dpf animals, *apoa4b.1, apoa4b.3, apoea, zgc:17,679,* and *gvinp1* were genes that were downregulated in *creb3l3* mutants in both feeding conditions. In adult animals, *clca1, cd8b,* and *ifi44f5* are upregulated in *creb3l3* mutants in both unfed and fed conditions. *apoa4b.2* is upregulated in unfed, adult *creb3l3* mutants, but downregulated after a HFM. *mmp17b, entpd8,* and *si:dkey-207m2.4* are upregulated in *creb3l3* mutants after a HFM but downregulated in unfed *creb3l3* mutants. *apoa4b.3* and *arpc1a* are downregulated in *creb3l3* mutant adults regardless of feeding. C: *plin2* expression fasted *creb3l3* adults. D: Expression of *apoea* and *apoeb* is downregulated in larval and adult *creb3l3* mutants. Creb3l3, cAMP-responsive element-binding protein 3–like 3; dpf, days post fertilization; Plin, perilipin.

We performed differentially expressed gene (DEG) analyses (Fig. 8A) between *creb3l3a*^*c802/c802*^*;creb3l3b*^*c803/c803*^ and *creb3l3a*^*c802/+*^*;creb3l3b*^*c803/+*^ siblings; analyzing gene expression at 6 dpf and adult stages. At 6 dpf, unfed *creb3l3* double mutants had 36 DEGs, while *creb3l3* double mutants after a HFM had 28 DEGs when compared to double heterozygous siblings. The comparison of DEGs between prandial states at 6 dpf revealed a failure to upregulate the expression of *apoa4b.1*, *apoa4b.3*, *apoea*, *gvinp1*, and the unannotated gene *zgc:17679* in *creb3l3a*^*c802/c802*^*;creb3l3b*^*c803/c803*^ mutants (Fig. 8B). As we saw in qRT-PCR experiments, the expression of *apoea, apoeb, apoa4a, apoa4b.1, apoa4b.2, and apoa4b.3* also fail to be induced in *creb3l3* mutants after a HFM (supplemental Fig. S5).

When we compared DEGs between fed and unfed adults, we found *clca1, cd8b,* and *ifi44f5* to be upregulated in *creb3l3* mutants in both feeding conditions. Many of the DEGs in both conditions of *creb3l3* mutant adults were immune response genes. *apoa4b.2* was upregulated in unfed, adult *creb3l3* mutants, but downregulated in *creb3l3* mutant adults after a HFM. Fasted Creb3l3-deficient adults display an increased expression of perilipin 2 (*plin2)* in the intestine (Fig. 8C). Plin proteins are lipid droplet–associated proteins that play a crucial role in regulating lipid metabolism by controlling the storage and hydrolysis of neutral lipids (51). We hypothesize that the increase in *plin2* expression is likely due to the aberrant accumulation of cytoplasmic lipid droplets in the mutant enterocytes. In the qRT-PCR studies, we showed that *creb3l3-*responsive genes are not induced in *creb3l3* mutants following an HFM. Our previous RNA-seq data in digestive tissues of wild-type animals (15) and our qRT-PCR data in *creb3l3a*^*c802/+*^*;creb3l3b*^*c803/+*^ (Fig. 4B, C) showed that *apoe* expression is upregulated following a meal. Although ApoE is currently not a known target of Creb3l3, the current RNA-seq analysis further confirms the downregulation of *apoea* and *apoeb* in *creb3l3* mutants in both larvae and adult animals either fasted or postprandial (Fig. 8D).

## Discussion

The intracellular processes governing the assembly, modification, and secretion of ApoB-lps by intestinal enterocytes remains poorly understood, however emerging evidence suggests that CREB3L3 may have a critical role in regulating these pathways. Humans with mutations in *CREB3L3* exhibit hypertriglyceridemia and hypercholesterolemia (13, 14). Previous studies have interrogated the mechanistic role of CREB3L3 by generating floxed mice to remove a large portion of *Creb3l3* (spanning intron 3 to intron 11) when used with tissue-specific Cre drivers (14). The liver-specific knockout of *Creb3l3* in mice exhibits hyperlipidemia and nonalcoholic steatohepatitis, while the intestine-specific *Creb3l3* knockout mice were not hyperlipidemic but did show a modest increase in the amount of total body fat (14). However, the underlying mechanism of this phenotype was unexplained.

To address major knowledge gaps in our understanding of the role of CREB3L3 in modulating dietary lipid physiology, we generated novel mutations in the two zebrafish orthologs of mammalian *CREB3L3 (creb3l3a* and *creb3l3b)*. In *creb3l3* double mutant zebrafish, we observed an accumulation of cytoplasmic lipid droplets in enterocytes which was fairly consistent with the retention of fat observed in the intestine-specific mouse model knockout; although, excess TGs were being stored in the zebrafish intestine, not in adipose tissue like in the intestine-specific *Creb3l3* knockout mice (14).

We were able to rule out the hypothesis that the increase in intestinal lipid observed *creb3l3* mutant zebrafish resulted from an increase in dietary lipid intake since mutant animals consumed similar amounts as control animals. Additionally, RNA-seq analysis revealed no evidence of an increase in expression of genes required for de novo lipogenesis. Interestingly, a large portion of the DEGs at 6 dpf in both fasted and fed states were involved in GTP binding activity, including the GTPase *gvinp1*, which may affect the regulation of vesicular trafficking.

During zebrafish larval development, 3 dpf is the period with the most ApoB-lp production, likely to support the rapid growth of the larvae during this time (24). In 3 dpf *creb3l3* mutants, there was a significant decrease of total number of ApoB-lps compared to control siblings. This finding suggests that the loss of Creb3l3 may slightly delay zebrafish larval development, which is also supported by our observation that *creb3l3* mutants still have yolk present at 6 dpf. However, *creb3l3* mutants were producing similar amounts of ApoB-lps by 5 and 6 dpf.

The rate of postprandial chylomicron secretion has been well studied in both mice and humans (52). To investigate the zebrafish postprandial plasma TG levels, we developed a strategy to gavage adult fish using a fluorescently labeled BODIPY fatty acid to quantify fluorescence in circulation (blood plasma) and intestinal tissue as a function of time. In postprandial gavage experiments using fluorescently labeled fatty acid, adult *creb3l3* mutants do not show evidence of increased lipid flux out of the adult intestine into the plasma. In fact, in the plasma of *creb3l3* mutant adults, we observed less fluorescent lipid compared to control siblings following a gavage of fluorescent lipid. These data could suggest that the newly consumed fluorescent dietary lipid is not being absorbed as readily in *creb3l3* mutants compared to controls. Though not statistically significant, *creb3l3* mutant adults did display a tendency toward reduced fluorescent lipid levels in the intestine, consistent with mutant animals absorbing less of the labeled lipid from the gavage. We know that juvenile *creb3l3* mutant animals have more retained enterocyte lipid that may inhibit dietary lipid absorption and subsequently attenuate ApoB-lp secretion. Additional studies will be needed to confirm whether more of the gavaged lipid is excreted from the intestine in *creb3l3* mutants, rather than being absorbed and secreted into circulation.

**Fig. 9:**
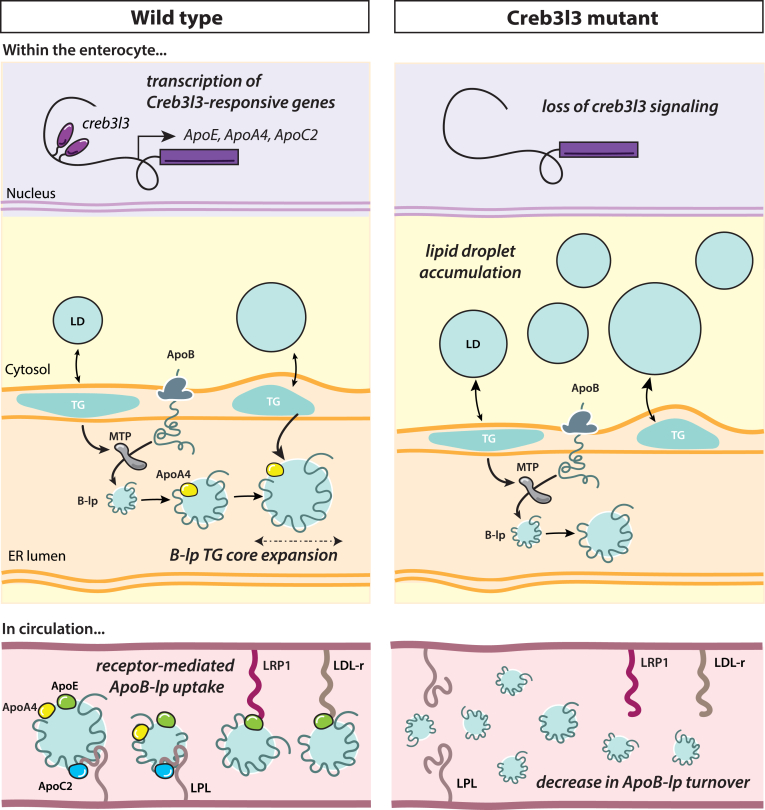
Summary of results and hypothetical model. Creb3l3 signaling activates Creb3l3-responsive genes such as ApoC2, ApoE, and ApoA4. We found that loss of Creb3l3 results in an increase of cytoplasmic lipid droplets in the enterocytes likely due to the loss of ApoA4. It is speculated that ApoA4 is responsible for expanding the size of the ApoB-lp through the addition of triglyceride potentially in poorly understood post ER steps. If enterocytes are unable to enlarge particles, the enterocyte may be directing excess lipid to cytoplasmic lipid droplets for storage. In circulation, ApoE facilitates the uptake of B-lp by peripheral tissues. In our study, we hypothesize that the increase of ApoB-lp half-life, or decrease of ApoB-lp turnover, is a direct result of the loss of ApoE from the ApoB-lp particle. ApoB-lp, apolipoprotein-B–containing lipoprotein; B-lp, ApoB-containing lipoprotein; Creb3l3, cAMP-responsive element-binding protein 3–like 3; ER, endoplasmic reticulum; LDL-r and LRP1, members low-density lipoprotein receptor family; LPL, lipoprotein lipase; MTP, microsomal triglyceride transfer protein; TG, triglyceride.

Our data indicate that *creb3l3* mutants are unable to increase the expression of specific apolipoprotein genes, specifically *apoa4*, which could be altering the balance between lipid storage and secretion within the enterocyte. ApoA4 is present in all vertebrates, highly expressed in both the intestine and liver, and strongly upregulated after a HFM (40). Loaded onto the nascent lipoprotein in the Golgi, ApoA4 is thought to facilitate the expansion of the neutral lipid core (53). After a HFM, we hypothesize that the loss of ApoA4 gene expression in *creb3l3* mutants may prevent the clearance of lipid from the enterocyte by reducing secretion of lipid in chylomicrons, thus shifting more lipid into storage in cytoplasmic lipid droplets (Fig. 9). Reduced lipidation of chylomicrons due to the reduction in ApoA4 could explain the lower lipid secretion rate from the intestine in *creb3l3* mutants.

The ApoA4 mouse knockout did not show any differences in lipid absorption or total TG secretion compared to wild-type mice (54). The overexpression of ApoA4 increased the rate of ApoB-lp secretion in rat epithelial cells (53). Paradoxically, the ApoA4 mouse knockout secreted larger chylomicrons from the intestine that were metabolized more slowly than chylomicrons in wild-type animals (55, 56).

Unfortunately, we were unable to directly test the size of chylomicrons in the *creb3l3* double mutant zebrafish because both of the ApoB reporter lines label ApoB-lps produced by both the intestine and the liver and are not specific to chylomicrons. Further, the native-PAGE ApoB-lp size gel assay has not been validated for quantifying chylomicrons, as we believe that they are so large they remain with tissue aggregates in the ZM band. It is also unclear whether the large deletion in the ApoA4 mouse knockout affected the expression of flanking genes in the same locus, including ApoA1 and ApoC3 on mouse chromosome 9 (57).

In zebrafish, the loss of Creb3l3 directly affects lipoprotein clearance, which is consistent with prior findings in ApoA4 knockout studies in mice (55, 58). Specifically, we found that ApoB-lps remain present in circulation for a longer amount of time in *creb3l3* mutants. Additionally, ApoE knockout mice also display a delay in lipoprotein clearance (59). ApoE is added to ApoB-lps in the Golgi and serves as a ligand to mediate the uptake of ApoB-lps by hepatic and other tissue receptors (Fig. 9) (60). Consistent with these findings, our data showing attenuated *apoe* expression in *creb3l3* mutants may indicate an important role for ApoE in ApoB-lp clearance. Unlike zebrafish, mammalian intestinal tissue expresses APOBEC which edits *apob* mRNA to produce ApoB-lps containing a truncated ApoB (ApoB-48) requiring ApoE for efficient liver clearance (61). Our data could suggest an unappreciated role for ApoE in the clearance of ApoB-lps containing full-length ApoB.

An additional finding supporting the shift between the synthesis of lipoproteins for secretion and cytoplasmic lipid droplets for storage in *creb3l3* mutants is the highly upregulated expression of *plin2* in fasted Creb3l3-deficient adults. *Plin2* is not a known Creb3l3 target gene, and increased *plin2* expression is unlikely to be the direct cause of lipid accumulation—rather, it appears to be a consequence of the aberrant cytoplasmic lipid droplet buildup in *creb3l3* mutant enterocytes. Further, we neither observe accumulation of ApoB-lps in the secretory pathway in enterocytes of *creb3l3* mutants (Fig. 3C) nor were the number of total ApoB-lp particles changed after 3 dpf. This data, together with the reduced postprandial plasma BODIPY-FA levels in *creb3l3* mutants fed a HFM (Fig. 7), suggests that the chylomicrons secreted by the intestine carry less lipid than those of the controls. This mechanism is supported by our failure to observe unsecreted ApoB-lps in enterocytes, our observation that total ApoB-lps particle number is not really changed after 3 dpf and that creb3l3 mutants fed a HFM have reduced postprandial plasma BODIPY-FA. The precise means by which lipids are partitioned into cytoplasmic lipid droplets versus lipoproteins remain unknown, but our data support a model in which Creb3l3 plays a regulatory role in this process.

Together, our results highlight the importance of Creb3l3 in modulating lipid clearance from the enterocyte by controlling ApoE and ApoA4 gene expression. Delayed lipid clearance from the bloodstream (loss of ApoE) may shift enterocyte activity from secreting lipoproteins (loss of ApoA4 regulating particle formation) to storing incoming dietary lipids as cytoplasmic lipid droplets.

## Data availability

The raw data and sequencing files supporting this study have been deposited with the *Journal of Lipid Research* and are available as supplementary files accompanying this article.

## Supplemental data

This article contains supplemental data.

## Conflict of interests

The authors declare that they have no conflicts of interest with the contents of this article.

## Supplementary Data

Larval_salmon.merged.gene

ADult_salmon.merged.gene
