# Supplementary material for: Creb3l3 deficiency promotes intestinal lipid accumulation and alters ApoB-containing lipoprotein kinetics
Source: J Lipid Res. 2025 May 29;66(7):100833. doi: 10.1016/j.jlr.2025.100833 (PMC12271627; doi:10.1016/j.jlr.2025.100833)
Supplement: Supplemental [file mmc3.docx]

Supplemental Table 1: CRISPR guide sequences.

| sgRNA | 5’ to 3’ Sequences |
| --- | --- |
| *creb3l3a* | taatacgactcactataGGTGGGCTTGCATTCGGTGGgttttagagctagaa |
| *creb3l3b* | taatacgactcactataGGAGAGCCGCAAGAAAAAGAgttttagagctagaa |
| chimeric sgRNA core sequence | AAAAGCACCGACTCGGTGCCACTTTTTCAAGTTGATAACGGACTAGCCTTATTTTAACTTGCTATTTCTAGCTCTAAAAC |

Supplemental Table 2: Genotyping primers.

| PCR Primer | 5’ to 3’ Sequences |
| --- | --- |
| *creb3l3a_fw* | ATTCCAGGCCGCGAAGGTGTCTCAGCGGTC |
| *creb3l3a_rv* | TTGATCACGGGAGGGAAGCA |
| *creb3l3b_fw* | AAGCAGTCGGCACAGGAGAGCCGCAAGAAG |
| *creb3l3b_rv* | CACTGGACTAAGCCAAAGGAAAATG |
| *apoBb.1_NanoLuc_fw* | GCTTCCTCTCCCATTTTTCC |
| *apoBb.1_NanoLuc_rv_1* | CCCCGAGATTCTGAAACAAAC |
| *apoBb.1_NanoLuc_rv_2* | AAGTGTCCATTGGCTTCGAT |
| *apoBB-.1_Dendra2_fw* | GCTTCCTCTCCCATTTTTCC |
| *apoBB-.1_Dendra2_rv* | ACCATGACCTTCGAGGACAA |

Supplemental Table 3. In situ probe design.

| *in situ* primers | 5’ to 3’ Sequences | Amplicon size (bp) |
| --- | --- | --- |
| *creb3l3a_fw* | TGGATCTCCTGTTTGGTCGC | 866 |
| *creb3l3a_rv* | GCGGGAGAATTTTGGCGTTT |  |
| *creb3l3b_fw* | CACAGAGCCGGATTTCTCCA | 756 |
| *creb3l3b_rv* | TGTGACTGGTCCATGTCTGC |  |

Supplemental Table 4: Quantitative Real-Time PCR Primer Sequences.

| Primer name | 5’ to 3’ oligo sequence |
| --- | --- |
| *apoa4a_fw* | GACCCAGCTCAAGCCTTATG |
| *apoa4a_rv* | GACCCAGCTCAAGCCTTATG |
| *apoa4b.1_fw* | GAGTTCCAGAAAACTGTGAGTCCTCTAGCT |
| *apoa4b.1_rv* | TCGTACAGAGAGATCAGCTGGTCTTTTAGG |
| *apoa4b.2_fw* | TTGTGGTCTTTGCACTTGCT |
| *apoa4b.2_rv* | TCATCTTGACGGTTTCCTCTG |
| *apoa4b.3_fw* | TGAAGGTTCTTGTGGTGCTC |
| *apoa4b.3_rv* | AATGGATTCCTCTGCGGTTT |
| *apobb.1_fw* | GCTTGAAGGAACCAGCAGTC |
| *apobb.1_rv* | AGTTGGTGGTTGGCATTAGC |
| *apoea_fw* | GAAGAGGCCGTGGATCAGTT |
| *apoea_rv* | GCTGTCAGAGATCAGTGTGTCTA |
| *apoeb_fw* | ACGCAAACTGAAGAAACGCC |
| *apoeb_rv* | TGTCCAGTAAAGGACAGTGC |
| *18S_fw* | TGCAGAACCCTCGCCAGTACAAAATCCCAG |
| *18S_rv* | CCAGAAGTGACGGAGACCACGGTGAGCCCT |


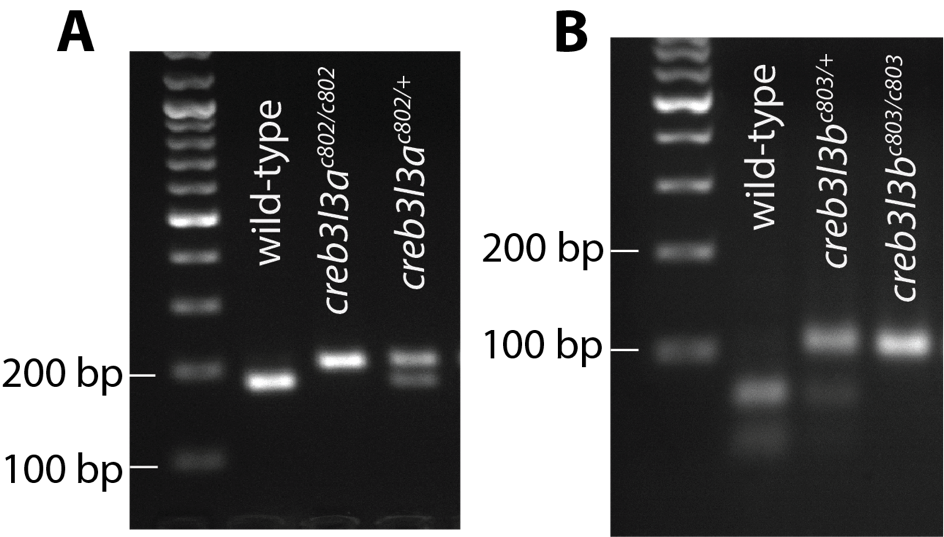


**Supplemental Figure 1: Agarose gel electrophoresis method to genotype *creb3l3* mutant alleles.** Electrophoresis of PCR products following a restriction enzyme digest. A) Genotyping gel for *creb3l3a^c802^* B) Genotyping gel for *creb3l3b^c803^*


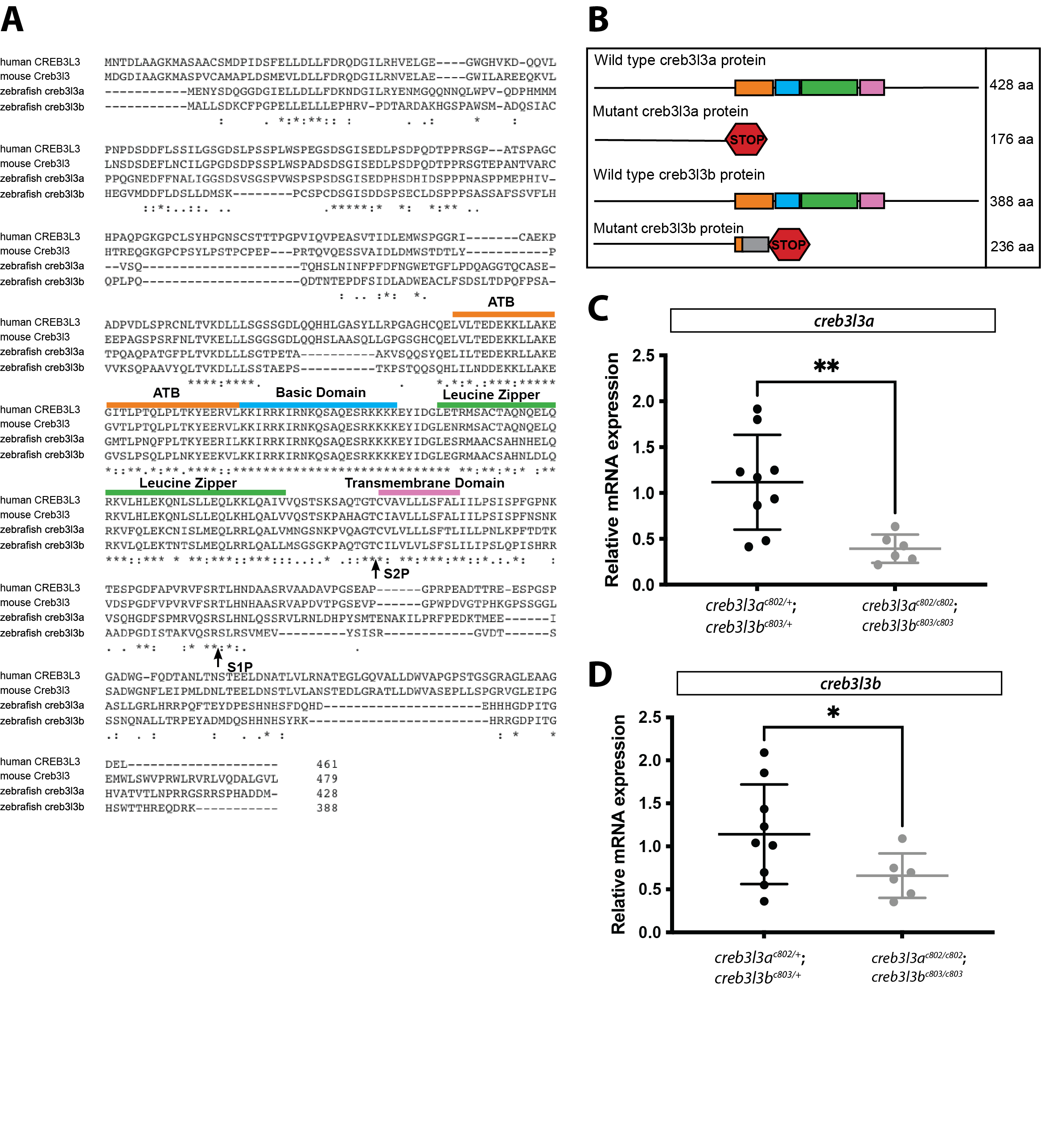


**Supplemental Figure 2: Amino Acid alignments of Creb3l3a and Creb3l3b.** A) Amino acid alignment of human Creb3l3, mouse Creb3l3, and zebrafish orthologs Creb3l3a and Creb3l3b. Domains of the transcription factor are mapped onto the amino acid sequence. (ATB: adjacent to basic leucine zipper (bZIP); S1P: site 1 protease; S2P: site 2 protease) B) Schematic of protein domains in the wild-type Creb3l3a and Creb3l3b. The mutant Creb3l3a protein contains a premature stop codon in the ATB domain. The mutant Creb3l3b protein contains a stop codon in the basic domain. Both mutant proteins are predicted to result in a truncated form of the protein. C) qPCR confirms downregulation of *creb3l3a* transcript in *creb3l3a^c802/c802^;creb3l3b^c803/c803^* 14 dpf animals. D) qPCR confirms downregulation of *creb3l3b* transcript in *creb3l3a^c802/c802^;creb3l3b^c803/c803^* 14 dpf animals. n = 6-9 individuals per genotype T-test with Welch’s correction ** p < 0.01, * p < 0.05

**
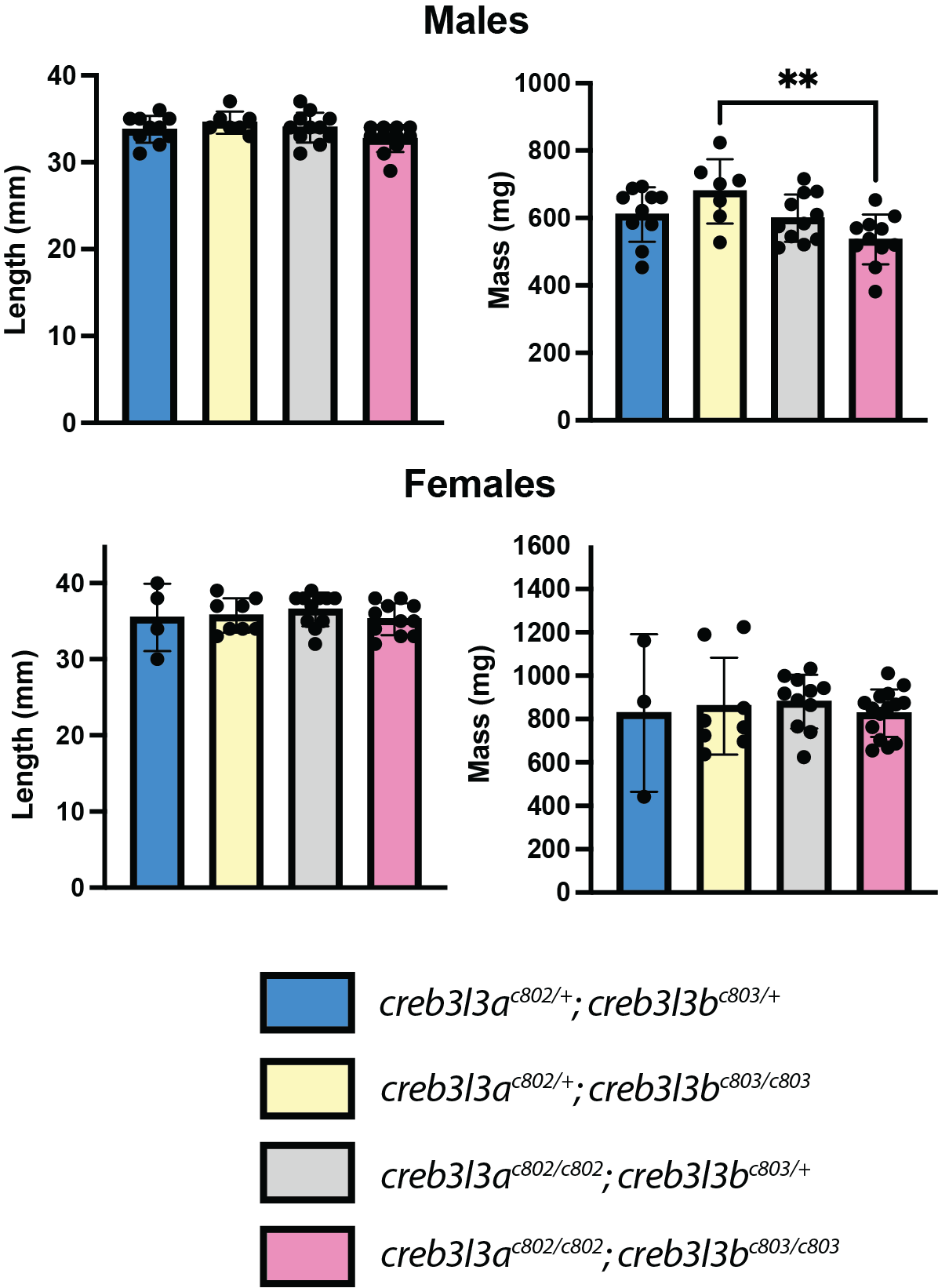

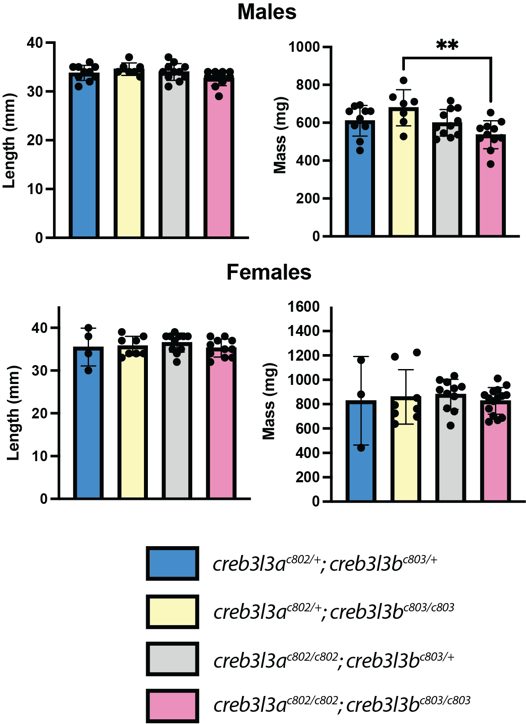
**

Supplemental Figure 3: *creb3l3* double homozygous mutants develop to adulthood similarly to double heterozygous control siblings. No differences between *creb3l3* double homozygous and double heterozygous siblings at 4 months post fertilization. *creb3l3a^c802/+^;creb3l3b^c803/c803^* males have slight, yet statistically significant increase in mass than *creb3l3a^c802/c802^;creb3l3b^c803/c803^* males. n = 3-14 individuals per genotype. One-way ANOVA with Brown-Forsythe test. ** pval < 0.01


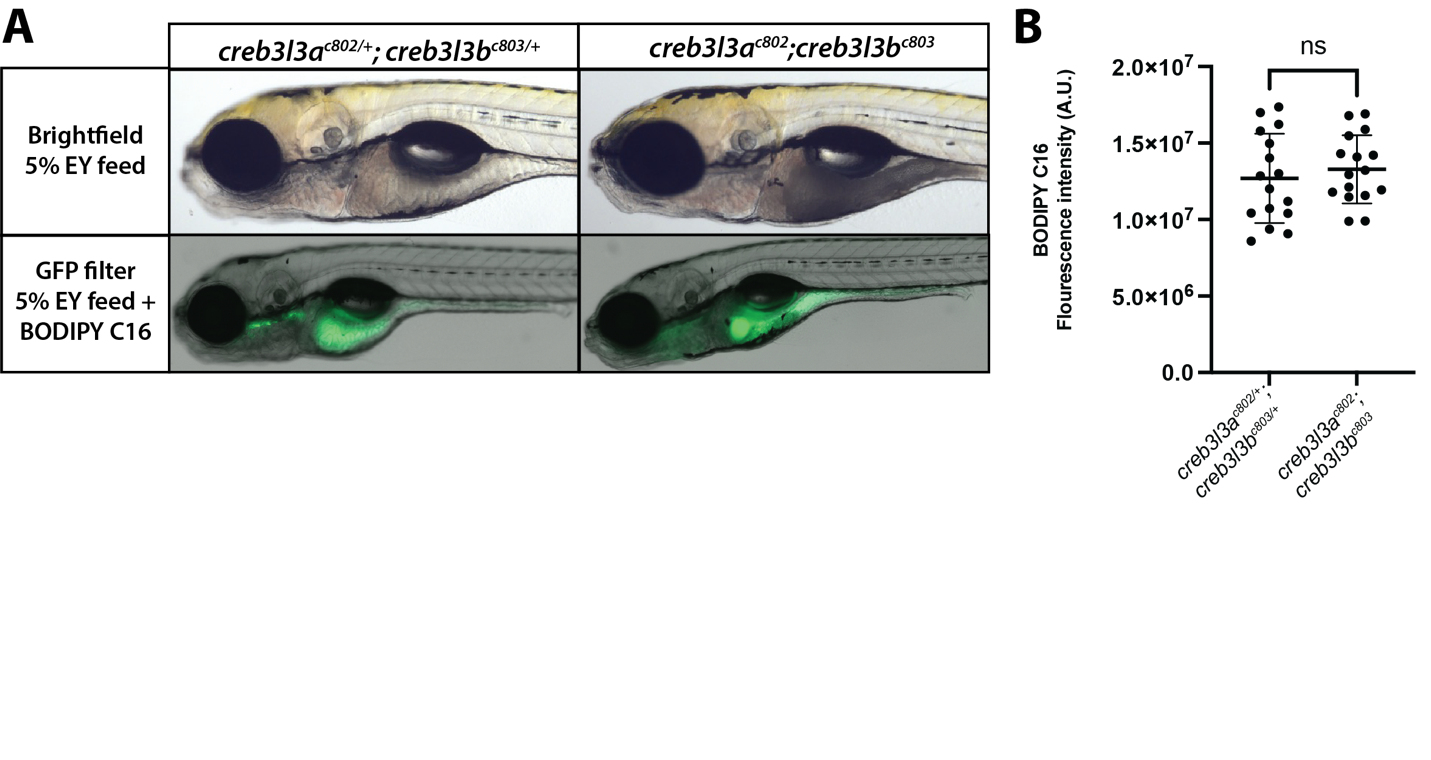


Supplemental Figure 4: Lipid accumulation in intestine of *creb3l3* double homozygous mutants is not due to increased consumption. A) 6 dpf *creb3l3* mutants after a 2-hour feed of a high-fat meal of 5% egg yolk with BODIPY-C16 followed by a 20-hour chase. Representative images under brightfield and GFP filter. B) Quantification of BODIPY C16 fluorescence immediately after high fat meal. No observed difference between genotypes of total BODIPY fluorescence. N = 3 clutches; 4-7 individuals per genotype per clutch, T-test. (BF, brightfield).


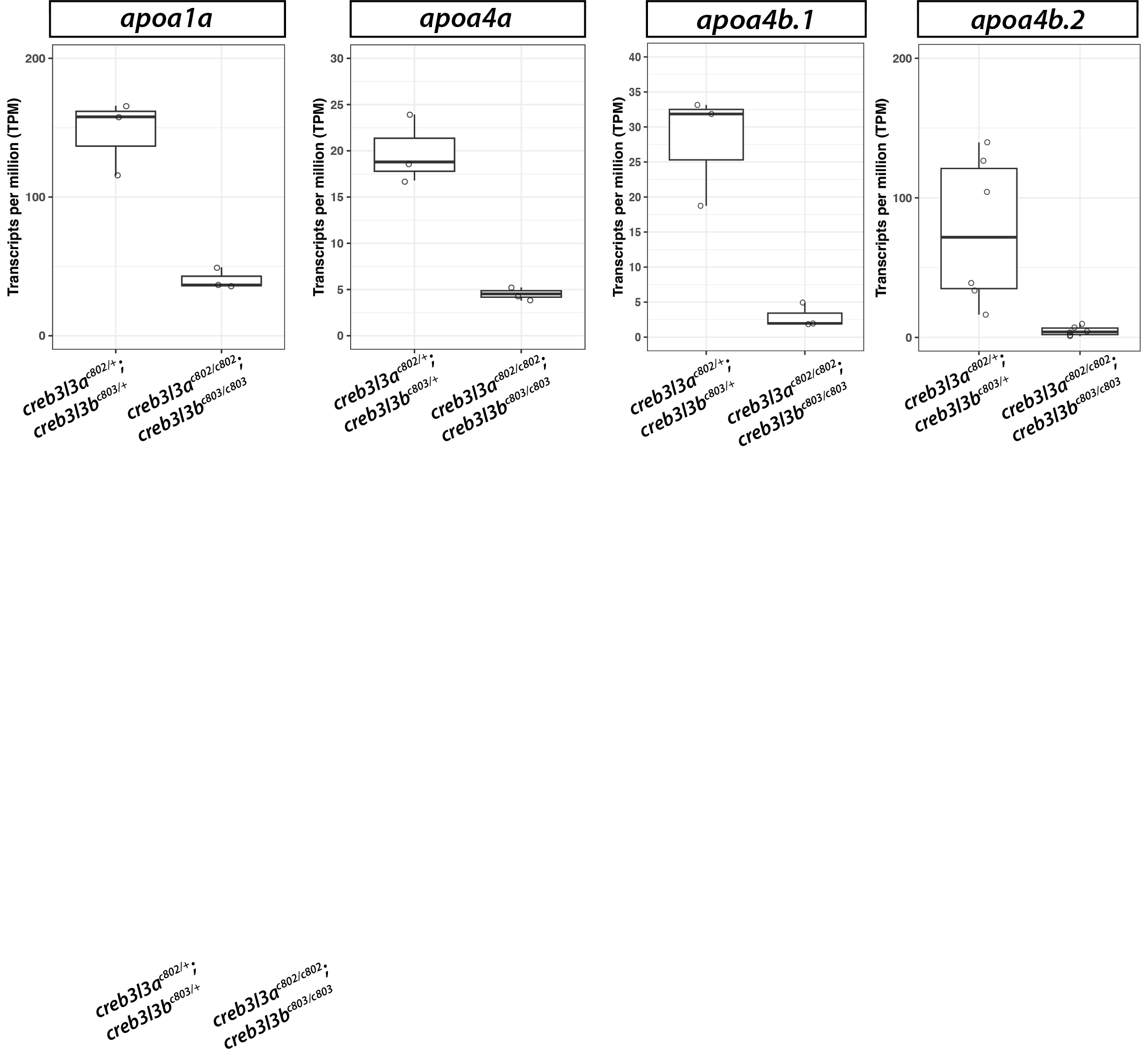


Supplemental Figure 5: Confirmation of downregulated Creb3l3 target genes after a high-fat meal (HFM). RNA-seq analysis from 6 dpf larvae confirms that *apoa1a, apoa4a, apoa4b.1,* and *apoa4b.2* are significantly downregulated in *creb3l3* mutants after a HFM.
